# Hitchhiking across continents: phoresy of pseudoscorpions (Arachnida, Pseudoscorpiones) on Diptera, with new European records

**DOI:** 10.3897/zookeys.1276.188186

**Published:** 2026-04-07

**Authors:** Jana Christophoryová, Vincent Mathy, Christoph Hörweg, Lucia Vičanová

**Affiliations:** 1 Department of Zoology, Faculty of Natural Sciences, Comenius University, Mlynská dolina, Ilkovičova 6, SK-842 15 Bratislava, Slovakia 3rd Zoological Department, Natural History Museum Vienna Vienna Austria; 2 Bénouville, France Faculty of Natural Sciences, Comenius University Bratislava Slovakia; 3 3rd Zoological Department, Natural History Museum Vienna, Burgring 7, A-1010 Vienna, Austria Unaffiliated Bénouville France

**Keywords:** Dispersal, distribution, fly, host specificity, phoront

## Abstract

Phoresy is an important dispersal strategy in pseudoscorpions, yet associations with Diptera have not been comprehensively revised for more than two decades. An updated synthesis of pseudoscorpion-Diptera phoresy is presented based on newly collected European material, a revision of previously studied Slovak material, a critical reassessment of worldwide published records, and available fossil evidence. In total, 11 previously unreported cases of pseudoscorpion phoresy from four European countries are documented, including the first records of *Dactylochelifer degeerii* (C.L. Koch, 1835) from France and of *Pselaphochernes scorpioides* (Hermann, 1804) from Slovenia. Seven dipteran species and three dipteran families are newly recorded worldwide as hosts of pseudoscorpion phoresy, and one case of multiple phoresy was recorded from Slovakia. Altogether, 172 records spanning the period from 1761 to 2025 were compiled and evaluated. Compared with previous syntheses, the number of known dipteran hosts was increased to 74 species from 30 families, and pseudoscorpion phoronts to 39 species from seven families. This study provides the most comprehensive overview of pseudoscorpion-Diptera phoresy to date and establishes a robust framework for future taxonomic, ecological, and evolutionary studies.

## Introduction

Even though arachnids are generally incapable of active long-distance dispersal, they occur in almost all terrestrial habitats. Their spread is primarily mediated by external vectors, with short-range dispersal facilitated by other animals and long-distance dispersal mainly achieved through air currents. Owing to these mechanisms, arachnids, together with insects, are often among the first animals to colonise newly available habitats (Szymkowiak et al. 2007). Dispersal strategies, such as phoresy or ballooning, are well-documented in spiders, mites, and pseudoscorpions and are typically associated with specific morphological and trophic adaptations, habitat preferences, and the availability and type of suitable carriers (Szymkowiak et al. 2007).

Phoresy is a dynamic, interspecific, and temporary relationship in which a phoront attaches to a host for the time required to migrate from one habitat to another, with dispersal being the primary outcome of this interaction (Camerik 2010). Records of phoretic pseudoscorpions date back to the eighteenth century (Poda 1761), yet the reasons underlying passive dispersal via phoresy in pseudoscorpions remain poorly known. Two hypotheses have been proposed to explain phoresy in pseudoscorpions (summarised in Poinar et al. 1998). The first hypothesis – that transport by other arthropods is accidental, motivated by hunger, and occurs because pseudoscorpions are incapable of consuming their hosts – had been rejected by Zeh and Zeh (1992b). The second hypothesis considers phoresy to be an adaptive behaviour functioning primarily for dispersal and is currently accepted. Phagophily does not represent the primary motivation for phoretic associations (Zeh and Zeh 1992c). Although some species may prey on mites present on their hosts or use host bodies as strategic sites for mating interactions, dispersal remains the principal function of phoresy in pseudoscorpions (Zeh and Zeh 1992c).

At a broader scale, phoresy has been reported across a wide range of animal taxa. A synthesis by Bartlow and Agosta (2021) showed that phoretic dispersal has been documented in animals belonging to 13 phyla, 25 classes, and 60 orders, predominantly involving small invertebrates. Fossil evidence, mainly preserved in amber, indicates that phoresy is an ancient phenomenon, with records particularly well documented for mites, springtails, and pseudoscorpions (Magowski 1995; Poinar et al. 1998; Penney et al. 2012). Some mite records date back approximately 85 million years (Magowski 1995), whereas pseudoscorpion phoresy is known from fossils up to 40 million years old (Poinar et al. 1998).

Within Pseudoscorpiones, phoresy has been documented in 12 of 26 recent families: Atemnidae, Cheiridiidae, Cheliferidae, Chernetidae, Chthoniidae, Garypinidae, Geogarypidae, Larcidae, Neobisiidae, Sternophoridae, Syarinidae, and Withiidae (e.g., Ressl 1965; Aguiar and Bührnheim 1998b; Poinar et al. 1998; Harms and Dunlop 2017), with Garypinidae restricted to fossil material. Phoretic pseudoscorpions have been reported from a wide range of hosts, including mammals, birds, ten insect orders (Coleoptera, Diptera, Hemiptera, Hymenoptera, Lepidoptera, Mecoptera, Neuroptera, Odonata, Orthoptera, and Plecoptera), as well as arachnids such as harvestmen, spiders, and scorpions (Aguiar and Bührnheim 1998a; Poinar et al. 1998; Harvey et al. 2015; Christophoryová et al. 2017b; Hetešová and Christophoryová 2022; Warburg et al. 2023b). Neuroptera were not included among phoretic hosts in the comprehensive overview by Poinar et al. (1998), but were subsequently reported by Aguiar and Bührnheim (1998b). As a result, Neuroptera are occasionally overlooked in later studies relying primarily on the earlier summary.

Among the various host groups, Diptera represent one of the most frequently reported phoretic hosts of pseudoscorpions (Poinar et al. 1998). This prominence can be attributed to their high abundance, ecological diversity, and frequent contact with microhabitats occupied by pseudoscorpions, such as decaying organic matter, tree hollows, bark, and nests. Many dipteran species are capable of active flight over considerable distances, making them effective vectors for passive dispersal. In addition, the apparent importance of Diptera as phoretic hosts is partly influenced by sampling bias, as flies are commonly collected using methods that increase the likelihood of detecting phoretic associations.

The most comprehensive synthesis of pseudoscorpion phoresy involving Diptera remains that of Poinar et al. (1998), which compiled records across all arthropod host groups. In the study, phoretic associations between pseudoscorpions and Diptera comprised 37 dipteran species (32 genera) from 17 families hosting 23 pseudoscorpion species (16 genera) from four families. Despite being more than a quarter of a century old, this work continues to be widely cited and serves as a primary reference for pseudoscorpion–Diptera phoretic associations. However, subsequent use of this compilation has revealed inconsistencies, duplications, and taxonomic inaccuracies, reflecting both the state of knowledge at the time and later advances in pseudoscorpion and dipteran systematics. Moreover, numerous records documented in earlier syntheses, such as Beier (1948), as well as data published after Poinar et al. (1998), were not included. These limitations highlight the need for an updated and critically revised synthesis.

During our research, we obtained valuable new material documenting pseudoscorpion phoresy on Diptera from Europe. Accordingly, we present a synthesis of pseudoscorpion–Diptera host–phoront associations based on (i) newly collected material, (ii) the revision of previously studied Slovak material, (iii) a critical reassessment of published records worldwide, and (iv) available fossil evidence.

## Materials and methods

### New material examined

**Figure 1.**
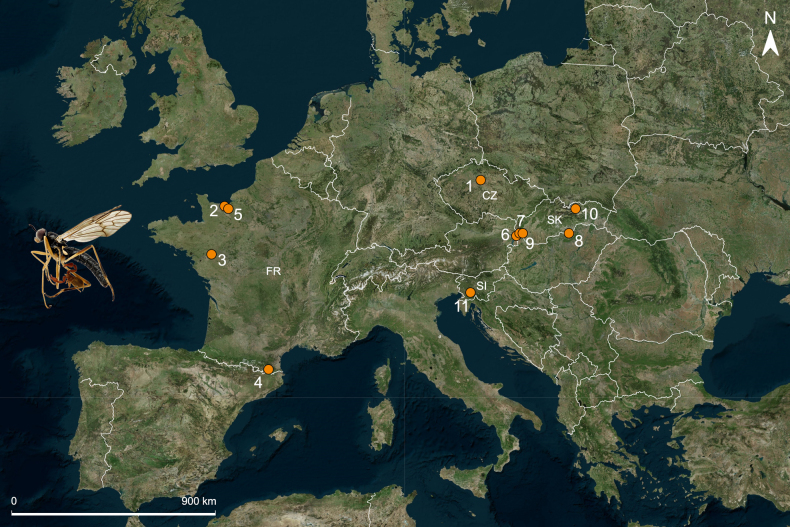
New records of pseudoscorpion phoresy in Europe. Locality codes are given in Materials and methods. Country abbreviations: CZ – Czechia, FR – France, SI – Slovenia, SK – Slovakia.

Pseudoscorpions were collected individually or using pyramid trap and Malaise traps at 11 localities in Czechia, France, Slovakia, and Slovenia (Fig. 1). Specimens were studied as temporary slide mounts prepared by immersion in lactic acid for clearing. After examination, they were rinsed in water and returned to 75% ethanol. All specimens were examined using a Leica DM1000 compound microscope with an ICC50 camera module (LAS EZ v. 3.4.0). Measurements were taken from digital images using AxioVision 40LE (v. 4.6.3.0). Digital photographs were taken using a Canon EOS 5D Mark II camera attached to a Zeiss Axio Zoom V16 stereomicroscope. Image stacks were created manually and merged using Zerene Stacker software (v.1.4). Spatial data used in the map were converted from original coordinates and visualised in QGIS (v. 3.36.2). Figures were edited in Adobe Photoshop CC (v. 25.6.0).

Pseudoscorpions were identified using the key in Krajčovičová et al. (2022) and data from Christophoryová et al. (2023). The latter study (Christophoryová et al. 2023) employed an interactive approach that combined molecular DNA barcoding, cytogenetic analysis, and multivariate statistical methods to investigate species boundaries within the genus *Lamprochernes*. These results are particularly relevant for specimens identified as *L. chyzeri* (Tömösváry, 1883), as the study revealed the presence of a cryptic species, *L. abditus* Christophoryová, Krajčovičová, Šťáhlavský, Španiel & Opatova, 2023, which is morphologically indistinguishable from *L. chyzeri* and can be reliably separated only using molecular and cytogenetic evidence. Because the examined specimens were not preserved in pure ethanol, molecular analyses could not be performed; therefore, the presence of *L. abditus* in the study area cannot be excluded. Consequently, specimens of this taxon in both the newly collected material and the revised Slovak material are herein referred to as *Lamprochernes cf. chyzeri*.

Several colleagues assisted with the identification of dipteran hosts and are acknowledged in the Results - New material examined section. One host specimen from locality 3 was identified only to the genus level because the material was poorly preserved and lacked diagnostic coloration. In some cases, the attachment site of phoretic pseudoscorpions was noted by the collector as the host’s leg; however, subsequent detachment of the specimens from the flies prevented more precise localisation of the attachment site on the leg (localities 3, 5, and 11).

Pseudoscorpions and the majority of Diptera hosts are deposited in the zoological collection of the Department of Zoology, Comenius University in Bratislava, Slovakia (depository codes FO 40/6–FO 50/6). Hosts from localities 1 and 8 are deposited in the Silesian Museum, Opava, Czechia.

### Revision of published data on phoresy from Slovakia

Published records on pseudoscorpion–dipteran phoresy from Slovakia (Mašán and Krištofík 1992; Christophoryová et al. 2011, 2017a, 2018, 2021; Červená et al. 2019; Hetešová and Christophoryová 2022) were revised in detail, as original material or supporting documentation was accessible to the authors. Several hosts, phoronts, or their sexes were additionally identified.

### Published data on the phoresy of Pseudoscorpiones and Diptera worldwide

Published data on the phoresy of Pseudoscorpiones and Diptera were reviewed from the earliest available records to the beginning of 2026. The literature survey was conducted with particular emphasis on two major revisionary works (Beier 1948; Poinar et al. 1998), which served as reference frameworks for reassessing earlier and subsequent records. In addition, all accessible publications were examined, including the catalogue (WPC 2026) as well as papers by key specialists in pseudoscorpion research, in order to retrieve all published information on phoresy.

The most recent comprehensive revision of published data on pseudoscorpion–dipteran phoresy was provided by Poinar et al. (1998). Their dataset, which summarised records by listing the host, phoront, continent, and literature source, was critically re-examined in the present study, with each original publication cited therein being individually reviewed, and the geographic information refined by replacing continent-level records with the precise country of occurrence.

Pseudoscorpion taxa were verified against WPC (2026), and issues related to synonymy, invalid names, and subsequent taxonomic revisions were resolved accordingly (see Notes).

Host identifications of Diptera were updated to their currently accepted names (see Notes), in many cases following consultation with dipteran specialists.

The same methodological approach was applied to the processing and evaluation of fossil data.

## Results and discussion

### New material examined

In total, 11 previously unreported cases of phoresy were documented in the present study from four European countries (Fig. 1; Table 1, data marked as “Present study”). The highest number of records was obtained from Slovakia (five) and France (four). Four pseudoscorpion species belonging to two families (Chernetidae and Cheliferidae) were found attached to the legs of 11 dipteran hosts representing nine families (Fanniidae, Limoniidae, Lonchaeidae, Muscidae, Scatopsidae, Sciomyzidae, Tipulidae, Ulidiidae, and Xylophagidae). One record from Slovakia involved multiple phoresy (Table 1). *Dactylochelifer degeerii* was recorded for the first time from France, and both the genus and species *Pselaphochernes scorpioides* were recorded for the first time from Slovenia. In addition, seven dipteran species were documented for the first time as hosts of pseudoscorpion phoresy (*Ceroxys urticae*, *Limonia nubeculosa*, *Neolimonia dumetorum*, *Pherbellia annulipes*, *Scatopse notata*, *Tipula vernalis*, *Xylophagus ater*) together with three dipteran families newly reported as hosts (Scatopsidae, Sciomyzidae, Xylophagidae) (Table 1).

**Table 1.** Extant records of phoresy between Diptera and Pseudoscorpiones.

Host taxa	Phoront taxa		Country	References
** Anthomyiidae **				
*Anthomyia pluvialis* (Linnaeus, 1758)	Chernetidae	*Lamprochernes cf. chyzeri* (Tömösváry, 1883)	SK	Hetešová and Christophoryová (2022)
*Anthomyia* Meigen, 1803	Chernetidae	*Lamprochernes nodosus* (Schrank, 1803)*^1^	DE	Gerstaecker (1862)
*Delia floralis* (Fallén, 1824)^2^	Chernetidae	*Allochernes peregrinus* Lohmander, 1939	SE	Lohmander (1939a, 1939b)
*Delia platura* (Meigen, 1826)	Chernetidae	*Lamprochernes nodosus* (Schrank, 1803)	SK	Christophoryová et al. (2018)
*Eutrichota affinis* (Stein, 1898)^3^	Chernetidae	*Pselaphochernes scorpioides* (Hermann, 1804)	US	Muchmore (1971)
*Eutrichota apicalis* (Stein, 1898)^4^	Chernetidae	*Hesperochernes* Chamberlin, 1924	US	Muchmore (1971)
** Calliphoridae **				
*Calliphora vicina* Robineau-Desvoidy, 1830*^5^	Chernetidae	*Lamprochernes nodosus* (Schrank, 1803)*^5^	DE	Leydig (1867, 1881, 1893)
*Calliphora vicina* Robineau-Desvoidy, 1830*^6^	Chernetidae	*Lamprochernes nodosus* (Schrank, 1803)^6^	GB-ENG	Pickard-Cambridge (1892); Kew (1901)
*Calliphora vomitoria* (Linnaeus, 1758)^7^	Cheliferidae	*Chelifer cancroides* (Linnaeus, 1758)^7^	GB-ENG	Donovan (1797)
*Calliphora vomitoria* (Linnaeus, 1758)^8^	Chernetidae	*Lamprochernes nodosus* (Schrank, 1803)*^8^	GB-ENG	Kirby and Spence (1826)
*Calliphora* Robineau-Desvoidy, 1830*^9^	Not specified	Not specified	AR	Berg (1893)
*Lucilia caesar* (Linnaeus, 1758)^10^	Chernetidae	*Lamprochernes nodosus* (Schrank, 1803)	SK	Mašán and Krištofík (1992)
*Lucilia sericata* (Meigen, 1826)	Chernetidae	*Lamprochernes nodosus* (Schrank, 1803)	ES	Castillo-Miralbes (2002)
*Stevenia* Robineau-Desvoidy, 1830^11^	Chernetidae	*Pselaphochernes lacertosus* (L. Koch, 1873)	ES	Zaragoza and Ruiz de la Cuesta Santiago (2017)
Not specified	Chernetidae	*Lamprochernes nodosus* (Schrank, 1803)	GB-ENG	Judson (1979)
** Chyromyidae **				
*Chyromya flava* (Linnaeus, 1758)	Chernetidae	*Lamprochernes cf. chyzeri* (Tömösváry, 1883)	SK	Hetešová and Christophoryová (2022)
** Clusiidae **				
*Clusiodes albimanus* (Meigen, 1830)	Chernetidae	*Pselaphochernes scorpioides* (Hermann, 1804)	SK	Hetešová and Christophoryová (2022)
** Culicidae **				
*Aedes sticticus* (Meigen, 1838)^12^	Chernetidae	*Lamprochernes nodosus* (Schrank, 1803)	CZ	Minář (1966)
*Anopheles freeborni* Aitken, 1939	Cheiridiidae	*Cheiridium* Menge, 1855	US-CA	Meyer et al. (1985)
*Culex tarsalis* Coquillett, 1896	Cheiridiidae	*Cheiridium* Menge, 1855	US-CA	Meyer et al. (1985)
Culicini [tribe] Meigen, 1818	Larcidae	*Larca lata* (Hansen, 1885)	AT	Ressl (1965)
Not specified	Chernetidae	Parachernes (Parachernes) inpai Mahnert, 1979	BR	Aguiar and Bührnheim (1998b)
Not specified	Larcidae	*Larca chamberlini* Benedict & Malcolm, 1978	US-CA	Benedict and Malcolm (1977)
** Dolichopodidae **				
*Dolichopus acuticornis* Wiedemann, 1817^13^	Syarinidae	*Syarinus strandi* (Ellingsen, 1901)^13^	FI	Kaisila (1949)
** Drosophilidae **				
*Drosophila funebris* (Fabricius, 1787)	Chernetidae	*Lamprochernes nodosus* (Schrank, 1803)	FR	Vachon (1940)
*Drosophila hydei* Sturtevant, 1921	Chernetidae	*Lamprochernes savignyi* (Simon, 1881)	AU	Harvey (1987)
*Drosophila melanogaster* Meigen, 1830	Chernetidae	*Lamprochernes savignyi* (Simon, 1881)	AU	Harvey (1987)
*Drosophila aff. repleta* Wollaston, 1858	Chernetidae	Parachernes (P.) beieri Muchmore, 1999^14^	EC	Mahnert (1987)
** Empididae **				
*Hilara* Meigen, 1822	Chernetidae	*Allochernes peregrinus* Lohmander, 1939	SK	Christophoryová et al. (2017a)
** Fanniidae **				
*Fannia canicularis* (Linnaeus, 1761)	Chernetidae	*Americhernes aff. incertus* Mahnert, 1979	BR	Lira et al. (2014)
*Fannia canicularis* (Linnaeus, 1761)*^15^	Chernetidae	*Lamprochernes nodosus* (Schrank, 1803)*^15^	GB-ENG	Macrae (1869)
*Fannia canicularis* (Linnaeus, 1761)^16^	Chernetidae	*Lamprochernes nodosus* (Schrank, 1803)*^16^	DE	Grimpe (1921)
*Fannia canicularis* (Linnaeus, 1761)^17^	Chernetidae	*Lamprochernes nodosus* (Schrank, 1803)^17^	Not specified	Berland (1932)
*Fannia canicularis* (Linnaeus, 1761)	Chernetidae	*Lamprochernes nodosus* (Schrank, 1803)	GB-ENG	Jones (1978)
*Fannia canicularis* (Linnaeus, 1761)	Chernetidae	*Lamprochernes savignyi* (Simon, 1881)	AU	Harvey (1987)
*Fannia canicularis* (Linnaeus, 1761)	Chernetidae	*Pselaphochernes scorpioides* (Hermann, 1804)	SK	Present study
*Fannia canicularis* (Linnaeus, 1761)	Chernetidae	*Sphenochernes camponoti* (Beier, 1970)	BR	Lira and Tizo-Pedroso (2017)
*Fannia pusio* (Wiedemann, 1830)	Chernetidae	*Sphenochernes camponoti* (Beier, 1970)	BR	Lira and Tizo-Pedroso (2017)
*Fannia yenhedi* Albuquerque, 1957	Chernetidae	*Sphenochernes camponoti* (Beier, 1970)	BR	Lira and Tizo-Pedroso (2017)
** Heleomyzidae **				
*Amoebaleria defessa* (Osten-Sacken, 1877)	Chernetidae	*Chelodamus* Chamberlin, 1925^18^	US	Muchmore (1971)
*Amoebaleria* Garrett, 1921^19^	Chernetidae	*Hesperochernes tamiae* Beier, 1930	US	Muchmore (1971)
*Scoliocentra*a Loew, 1862	Not specified	Not specified	N America	Marshall (2012)
*Tephrochlamys rufiventris* (Meigen, 1830)*^20^	Chernetidae	*Lamprochernes nodosus* (Schrank, 1803)*^20^	FR	Burr (1919)
** Hybotidae **				
*Leptopeza flavipes* (Meigen, 1820)	Chernetidae	*Pselaphochernes scorpioides* (Hermann, 1804)	SK	Červená et al. (2019)
*Leptopeza flavipes* (Meigen, 1820)	Chernetidae	*Pselaphochernes scorpioides* (Hermann, 1804)	AT	Červená et al. (2019)
*Leptopeza flavipes* (Meigen, 1820)	Chernetidae	*Pselaphochernes scorpioides* (Hermann, 1804)	AT	Červená et al. (2019)
** Limoniidae **				
*Achyrolimonia decemmaculata* (Loew, 1873)^21^	Chernetidae	*Pselaphochernes scorpioides* (Hermann, 1804)	GB-ENG	Hinton (1954)
*Achyrolimonia decemmaculata* (Loew, 1873)^22^	Chernetidae	*Pselaphochernes scorpioides* (Hermann, 1804)	GB-ENG	Jones (1978)
*Achyrolimonia decemmaculata* (Loew, 1873)	Chernetidae	*Pselaphochernes scorpioides* (Hermann, 1804)	ES	Mederos and Zaragoza (2017)
*Limonia nubeculosa* Meigen, 1804	Chernetidae	*Pselaphochernes scorpioides* (Hermann, 1804)	SI	Present study
*Neolimonia dumetorum* (Meigen, 1804)	Chernetidae	*Pselaphochernes scorpioides* (Hermann, 1804)	FR	Present study
Not specified	Cheliferidae	*Dactylochelifer degeerii* (C.L. Koch, 1835)^23^	AT	Ressl (1995)
Not specified	Not specified	Not specified	NL	Jong et al. (2024)
** Lonchaeidae **				
*Lonchaea carpathica* Kovalev, 1974	Chernetidae	*Chernes cimicoides* (Fabricius, 1793)	CZ	Christophoryová and Blažej (2025)
*Lonchaea chorea* (Fabricius, 1781)	Chernetidae	*Lamprochernes chyzeri* (Tömösváry, 1883)	GB-ENG	Jones (1978)
*Lonchaea chorea* (Fabricius, 1781)	Chernetidae	*Lamprochernes cf. chyzeri* (Tömösváry, 1883)	SK	Present study
*Lonchaea chorea* (Fabricius, 1781)^24^	Chernetidae	*Lamprochernes nodosus* (Schrank, 1803)^24^	GB-ENG	Ed (1834)
*Lonchaea chorea* (Fabricius, 1781)^25^	Chernetidae	*Lamprochernes nodosus* (Schrank, 1803)^25^	GB-ENG	Graham-Smith (1916)
*Lonchaea chorea* (Fabricius, 1781)	Chernetidae	*Lamprochernes nodosus* (Schrank, 1803)	GB-ENG	Jones (1978)
*Lonchaea laticornis* Meigen, 1826	Cheliferidae	*Rhacochelifer similis* Beier, 1932	TN	Vachon (1940)
*Lonchaea* Fallén, 1820	Chernetidae	*Lamprochernes cf. chyzeri* (Tömösváry, 1883)	SK	Christophoryová et al. (2021)
*Lonchaea* Fallén, 1820	Chernetidae	*Lamprochernes nodosus* (Schrank, 1803)	GB-ENG	Jones (1978)
*Lonchaea* Fallén, 1820	Chernetidae	*Lamprochernes nodosus* (Schrank, 1803)	GB-ENG	Judson (1979)
*Lonchaea* Fallén, 1820	Chernetidae	*Lamprochernes savignyi* (Simon, 1881)^26^	GB-ENG	Jones (1978)
** Micropezidae **				
*Grallipeza gracilis* Hennig, 1934	Not specified	Not specified	EC	Marshall (2012)
*Rainieria calceata* (Fallén, 1820)	Chernetidae	*Lamprochernes chyzeri* (Tömösváry, 1883)	CH	DeVore-Scribante (1999)
*Scipopus* Enderlein, 1922	Chernetidae	*Americhernes oblongus* (Say, 1821)	PA	Martínez et al. (2020)
Not specified	Not specified	Not specified	CR	Szymkowiak et al. (2007)
** Muscidae **				
*Hydrotaea ignava* (Harris, 1780)^27^	Chernetidae	*Lamprochernes nodosus* (Schrank, 1803)^27^	GB-ENG	Graham-Smith (1916)
*Hydrotaea meteorica* (Linnaeus, 1758)^28^	Chernetidae	*Lamprochernes nodosus* (Schrank, 1803)^28^	GB-ENG	Anonymous (1834)
*Hydrotaea similis* Meade, 1887	Chernetidae	*Lamprochernes nodosus* (Schrank, 1803)	SK	Mašán and Krištofík (1992)
*Musca autumnalis* De Geer, 1776	Chernetidae	*Lamprochernes cf. chyzeri* (Tömösváry, 1883)	SK	Present study
*Musca autumnalis* De Geer, 1776^29^	Chernetidae	*Lamprochernes nodosus* (Schrank, 1803)^29^	GB-ENG	Graham-Smith (1916)
*Musca autumnalis* De Geer, 1776	Chernetidae	*Lamprochernes nodosus* (Schrank, 1803)	GB-ENG	Jones (1978)
*Musca domestica* Linnaeus, 1758	Cheliferidae	*Chelifer cancroides* (Linnaeus, 1758)	Not specified	Anonymous (1879)
*Musca domestica* Linnaeus, 1758*^30^	Cheliferidae	*Chelifer cancroides* (Linnaeus, 1758)	ES	Franganillo (1913)
*Musca domestica* Linnaeus, 1758	Cheliferidae	*Chelifer cancroides* (Linnaeus, 1758)	Not specified	Beier (1951)
*Musca domestica* Linnaeus, 1758	Cheliferidae	*Chelifer cancroides* (Linnaeus, 1758)	IE	Jones (1978)
*Musca domestica* Linnaeus, 1758	Chernetidae	*Chernes cimicoides* (Fabricius, 1793)	CZ	Stecker (1875)
*Musca domestica* Linnaeus, 1758	Chernetidae	*Dinocheirus panzeri* (C.L. Koch, 1836)^31^	BE	Preudhomme de Borre (1873)
*Musca domestica* Linnaeus, 1758	Chernetidae	*Dinocheirus panzeri* (C.L. Koch, 1836)^32^	AT	Ressl (1965)
*Musca domestica* Linnaeus, 1758	Chernetidae	*Lamprochernes chyzeri* (Tömösváry, 1883)	DK	Meinertz (1964)
*Musca domestica* Linnaeus, 1758	Chernetidae	*Lamprochernes cf. chyzeri* (Tömösváry, 1883)	SK	Christophoryová et al. (2011)
*Musca domestica* Linnaeus, 1758*^33^	Chernetidae	*Lamprochernes nodosus* (Schrank, 1803)^33^	AT	Poda (1761)
*Musca domestica* Linnaeus, 1758*^34^	Chernetidae	*Lamprochernes nodosus* (Schrank, 1803)*^34^	GB-ENG	Adams (1787)
*Musca domestica* Linnaeus, 1758*^35^	Chernetidae	*Lamprochernes nodosus* (Schrank, 1803)^35^	Europe	Hermann (1804)
*Musca domestica* Linnaeus, 1758*^36^	Chernetidae	*Lamprochernes nodosus* (Schrank, 1803)*^36^	GB-ENG	Leach (1817)
*Musca domestica* Linnaeus, 1758^37^	Chernetidae	*Lamprochernes nodosus* (Schrank, 1803)*^37^	GB-ENG	Anonymous (1831, 1832)
*Musca domestica* Linnaeus, 1758	Chernetidae	*Lamprochernes nodosus* (Schrank, 1803)^38^	GB-ENG	Anonymous (1834)
*Musca domestica* Linnaeus, 1758	Chernetidae	*Lamprochernes nodosus* (Schrank, 1803)^38^	GB-ENG	Lukis (1834)
*Musca domestica* Linnaeus, 1758	Chernetidae	*Lamprochernes nodosus* (Schrank, 1803)^38^	GB-ENG	Moore (1834)
*Musca domestica* Linnaeus, 1758	Chernetidae	*Lamprochernes nodosus* (Schrank, 1803)*^1^	GB-ENG	Clarke (1858)
*Musca domestica* Linnaeus, 1758	Chernetidae	*Lamprochernes nodosus* (Schrank, 1803)*^39^	GB-ENG	Stainton (1865)
*Musca domestica* Linnaeus, 1758	Chernetidae	*Lamprochernes nodosus* (Schrank, 1803)^40^	GB-ENG	Stevens (1866)
*Musca domestica* Linnaeus, 1758	Chernetidae	*Lamprochernes nodosus* (Schrank, 1803)^40^	GB-ENG	Stevens (1866)
*Musca domestica* Linnaeus, 1758*^36^	Chernetidae	*Lamprochernes nodosus* (Schrank, 1803)*^36^	GB-ENG	M’Intire (1869)
*Musca domestica* Linnaeus, 1758	Chernetidae	*Lamprochernes nodosus* (Schrank, 1803)*^41^	GB-ENG	Bisshopp (1871)
*Musca domestica* Linnaeus, 1758	Chernetidae	*Lamprochernes nodosus* (Schrank, 1803)^42^	CH, DE, FR	Koch (1873)
*Musca domestica* Linnaeus, 1758*^43^	Chernetidae	*Lamprochernes nodosus* (Schrank, 1803)^43^	GB-ENG	Anonymous (1875); Newman (1875)
*Musca domestica* Linnaeus, 1758*^44^	Chernetidae	*Lamprochernes nodosus* (Schrank, 1803)^44^	GB-ENG	Dale (1878)
*Musca domestica* Linnaeus, 1758*^45^	Chernetidae	*Lamprochernes nodosus* (Schrank, 1803)^45^	FR	Simon (1879)
*Musca domestica* Linnaeus, 1758*^46^	Chernetidae	*Lamprochernes nodosus* (Schrank, 1803)^46^	FR	Courtois de Langlade (1883); Mégnin (1886)
*Musca domestica* Linnaeus, 1758	Chernetidae	*Lamprochernes nodosus* (Schrank, 1803)^47^	GB-ENG	Pickard-Cambridge (1885)
*Musca domestica* Linnaeus, 1758*^48^	Chernetidae	*Lamprochernes nodosus* (Schrank, 1803)^48^	GB-ENG	Pickard-Cambridge (1892)
*Musca domestica* Linnaeus, 1758	Chernetidae	*Lamprochernes nodosus* (Schrank, 1803)^38^	DE	Hess (1894)
*Musca domestica* Linnaeus, 1758	Chernetidae	*Lamprochernes nodosus* (Schrank, 1803)^49^	CH	Müller and Schenkel (1895); Lessert (1911)
*Musca domestica* Linnaeus, 1758	Chernetidae	*Lamprochernes nodosus* (Schrank, 1803)^38^	IT	Fenizia (1902)
*Musca domestica* Linnaeus, 1758	Chernetidae	*Lamprochernes nodosus* (Schrank, 1803)*^50^	GB-ENG	Hill (1905); Hickson (1905)
*Musca domestica* Linnaeus, 1758	Chernetidae	*Lamprochernes nodosus* (Schrank, 1803)^47^	GB-ENG	Pocock (1905)
*Musca domestica* Linnaeus, 1758	Chernetidae	*Lamprochernes nodosus* (Schrank, 1803)^51^	CH	Frey-Gessner (1906); Lessert (1911)
*Musca domestica* Linnaeus, 1758	Chernetidae	*Lamprochernes nodosus* (Schrank, 1803)^47^	GB-ENG	Jackson (1907)
*Musca domestica* Linnaeus, 1758*^45^	Chernetidae	*Lamprochernes nodosus* (Schrank, 1803)^45^	DE	Tullgren (1907)
*Musca domestica* Linnaeus, 1758*^45^	Chernetidae	*Lamprochernes nodosus* (Schrank, 1803)^45^	DE	Ellingsen (1908)
*Musca domestica* Linnaeus, 1758	Chernetidae	*Lamprochernes nodosus* (Schrank, 1803)^47^	GB-ENG	Jackson (1908)
*Musca domestica* Linnaeus, 1758*^48^	Chernetidae	*Lamprochernes nodosus* (Schrank, 1803)^48^	GB-SCT	Godfrey (1909)
*Musca domestica* Linnaeus, 1758	Chernetidae	*Lamprochernes nodosus* (Schrank, 1803)^47^	GB-ENG	Hewitt (1910, 1914)
*Musca domestica* Linnaeus, 1758	Chernetidae	*Lamprochernes nodosus* (Schrank, 1803)^52^	CH	Lessert (1911)
*Musca domestica* Linnaeus, 1758*^53^	Chernetidae	*Lamprochernes nodosus* (Schrank, 1803)^53^	IE ^53^	Carpenter (1912)
*Musca domestica* Linnaeus, 1758*^45^	Chernetidae	*Lamprochernes nodosus* (Schrank, 1803)^45^	GB-ENG	Ellingsen (1913)
*Musca domestica* Linnaeus, 1758	Chernetidae	*Lamprochernes nodosus* (Schrank, 1803)^47^	GB-ENG	Graham-Smith (1916)
*Musca domestica* Linnaeus, 1758	Chernetidae	*Lamprochernes nodosus* (Schrank, 1803)^47^	GB-ENG	Graham-Smith (1916)
*Musca domestica* Linnaeus, 1758	Chernetidae	*Lamprochernes nodosus* (Schrank, 1803)^47^	GB-ENG	Graham-Smith (1916)
*Musca domestica* Linnaeus, 1758	Chernetidae	*Lamprochernes nodosus* (Schrank, 1803)^47^	GB-ENG	Graham-Smith (1916)
*Musca domestica* Linnaeus, 1758	Chernetidae	*Lamprochernes nodosus* (Schrank, 1803)^54^	ES	Navás (1918)
*Musca domestica* Linnaeus, 1758	Chernetidae	*Lamprochernes nodosus* (Schrank, 1803)^55^	FR	Burr (1919)
*Musca domestica* Linnaeus, 1758	Chernetidae	*Lamprochernes nodosus* (Schrank, 1803)*^41^	DE	Grimpe (1921)
*Musca domestica* Linnaeus, 1758	Chernetidae	*Lamprochernes nodosus* (Schrank, 1803)*^41^	DE	Grimpe (1921)
*Musca domestica* Linnaeus, 1758	Chernetidae	*Lamprochernes nodosus* (Schrank, 1803)*^41^	DE	Grimpe (1921)
*Musca domestica* Linnaeus, 1758	Chernetidae	*Lamprochernes nodosus* (Schrank, 1803)*^41^	DE	Grimpe (1921)
*Musca domestica* Linnaeus, 1758	Chernetidae	*Lamprochernes nodosus* (Schrank, 1803)*^41^	DE	Grimpe (1921)
*Musca domestica* Linnaeus, 1758	Chernetidae	*Lamprochernes nodosus* (Schrank, 1803)*^41^	DE	Grimpe (1921)
*Musca domestica* Linnaeus, 1758	Chernetidae	*Lamprochernes nodosus* (Schrank, 1803)*^41^	DE	Grimpe (1921)
*Musca domestica* Linnaeus, 1758	Chernetidae	*Lamprochernes nodosus* (Schrank, 1803)^54^	IT	Caporiacco (1923)
*Musca domestica* Linnaeus, 1758*^45^	Chernetidae	*Lamprochernes nodosus* (Schrank, 1803)^45^	ES	Navás (1925)
*Musca domestica* Linnaeus, 1758	Chernetidae	*Lamprochernes nodosus* (Schrank, 1803)^52^	IT	Beier (1929)
*Musca domestica* Linnaeus, 1758	Chernetidae	*Lamprochernes nodosus* (Schrank, 1803)^56^	FR	Berland (1932)
*Musca domestica* Linnaeus, 1758*^30^	Chernetidae	*Lamprochernes nodosus* (Schrank, 1803)	FR	Vachon (1934a)
*Musca domestica* Linnaeus, 1758	Chernetidae	*Lamprochernes nodosus* (Schrank, 1803)	FR	Vachon (1934b)
*Musca domestica* Linnaeus, 1758	Chernetidae	*Lamprochernes nodosus* (Schrank, 1803)^54^	former USSR	Redikorzev (1936)
*Musca domestica* Linnaeus, 1758	Chernetidae	*Lamprochernes nodosus* (Schrank, 1803)	AT	Beier (1939)
*Musca domestica* Linnaeus, 1758	Chernetidae	*Lamprochernes nodosus* (Schrank, 1803)	SE	Lohmander (1939a)
*Musca domestica* Linnaeus, 1758	Chernetidae	*Lamprochernes nodosus* (Schrank, 1803)	SE	Lohmander (1939a)
*Musca domestica* Linnaeus, 1758	Chernetidae	*Lamprochernes nodosus* (Schrank, 1803)	AT	Beier (1948)
*Musca domestica* Linnaeus, 1758	Chernetidae	*Lamprochernes nodosus* (Schrank, 1803)	KG	Redikorzev (1949)
*Musca domestica* Linnaeus, 1758	Chernetidae	*Lamprochernes nodosus* (Schrank, 1803)	FR	Vachon (1953)
*Musca domestica* Linnaeus, 1758	Chernetidae	*Lamprochernes nodosus* (Schrank, 1803)	FR	Vachon (1954)
*Musca domestica* Linnaeus, 1758	Chernetidae	*Lamprochernes nodosus* (Schrank, 1803)	AT	Ressl and Beier (1958)
*Musca domestica* Linnaeus, 1758	Chernetidae	*Lamprochernes nodosus* (Schrank, 1803)	AT	Ressl (1965)
*Musca domestica* Linnaeus, 1758	Chernetidae	*Lamprochernes nodosus* (Schrank, 1803)	GB-ENG	Jones (1970)
*Musca domestica* Linnaeus, 1758	Chernetidae	*Lamprochernes nodosus* (Schrank, 1803)	AT	Ressl (1970)
*Musca domestica* Linnaeus, 1758	Chernetidae	*Lamprochernes nodosus* (Schrank, 1803)	GB-ENG	Jones (1978)
*Musca domestica* Linnaeus, 1758	Chernetidae	*Lamprochernes nodosus* (Schrank, 1803)	GB-ENG	Judson (1979)
*Musca domestica* Linnaeus, 1758	Chernetidae	*Lamprochernes nodosus* (Schrank, 1803)	DK	Andersen (1987)
*Musca domestica* Linnaeus, 1758	Chernetidae	*Lamprochernes nodosus* (Schrank, 1803)	DK	Andersen (1987)
*Musca domestica* Linnaeus, 1758	Chernetidae	*Lamprochernes nodosus* (Schrank, 1803)	DK	Andersen (1987)
*Musca domestica* Linnaeus, 1758	Chernetidae	*Lamprochernes nodosus* (Schrank, 1803)	DE	Carl (1994)
*Musca domestica* Linnaeus, 1758	Chernetidae	*Lamprochernes nodosus* (Schrank, 1803)	DE	Bellmann (1997)
*Musca domestica* Linnaeus, 1758	Chernetidae	*Lamprochernes nodosus* (Schrank, 1803)	SK	Christophoryová et al. (2017a)
*Musca domestica* Linnaeus, 1758	Chernetidae	*Lamprochernes savignyi* (Simon, 1881)^57^	IE	Stephens (1910); Kew (1916)
*Musca domestica* Linnaeus, 1758	Chernetidae	*Lamprochernes savignyi* (Simon, 1881)^58^	IE ^58^	Kew (1916)
*Musca domestica* Linnaeus, 1758^59^	Chernetidae	*Lamprochernes savignyi* (Simon, 1881)^59^	JP	Morikawa (1960)
*Musca domestica* Linnaeus, 1758	Chernetidae	*Lamprochernes savignyi* (Simon, 1881)	AU	Harvey (1987)
*Musca domestica* Linnaeus, 1758	Chernetidae	*Lamprochernes savignyi* (Simon, 1881)	AU	Harvey (1987)
*Musca domestica* Linnaeus, 1758	Chernetidae	*Lamprochernes* Tömösváry, 1883^60^	US	Muchmore (1971)
*Musca domestica* Linnaeus, 1758	Neobisiidae	Neobisium (Neobisium) carcinoides (Hermann, 1804)^61^	GB-ENG	Jackson (1907)
*Musca domestica* Linnaeus, 1758	Neobisiidae	Neobisium (N.) sylvaticum (C.L. Koch, 1835)	Europe	Poinar et al. (1998)
*Musca domestica* Linnaeus, 1758	Chernetidae	*Pselaphochernes lacertosus* (L. Koch, 1873)	ES	Domínguez et al. (2008)
*Musca domestica* Linnaeus, 1758	Chernetidae	*Pselaphochernes scorpioides* (Hermann, 1804)	GB-ENG	Jones (1978)
*Musca domestica* Linnaeus, 1758	Not specified	Not specified^62^	US-PA	Leidy (1877); Muchmore (1971)
*Musca domestica* Linnaeus, 1758	Not specified	Not specified^63^	N America	Webster (1897); Muchmore (1971)
*Musca domestica* Linnaeus, 1758	Not specified	Not specified	NZ	Edwards (1955)
*Musca* Linnaeus, 1758	Chernetidae	*Lamprochernes savignyi* (Simon, 1881)	ZA	Beier (1953)
*Musca*? Linnaeus, 1758	Chernetidae	*Pselaphochernes* Beier, 1932	US	Muchmore (1971)
*Muscina stabulans* (Fallén, 1817)	Chernetidae	*Pselaphochernes scorpioides* (Hermann, 1804)	FR	Vachon (1953)
Not specified	Chernetidae	*Dinocheirus serratus* (Moles, 1914)^64^	US-CA	Chamberlin (1952)
Not specified^65^	Chernetidae	*Lamprochernes nodosus* (Schrank, 1803)^65^	GH ^65^	Ellingsen (1913)
*Stomoxys calcitrans* (Linnaeus, 1758)	Chernetidae	*Lamprochernes cf. chyzeri* (Tömösváry, 1883)	SK	Christophoryová et al. (2017a)
*Stomoxys calcitrans* (Linnaeus, 1758)^66^	Chernetidae	*Lamprochernes nodosus* (Schrank, 1803)^66^	GB-ENG	Graham-Smith (1916)
*Stomoxys calcitrans* (Linnaeus, 1758)	Chernetidae	*Lamprochernes nodosus* (Schrank, 1803)	FR	Vachon (1940)
*Stomoxys calcitrans* (Linnaeus, 1758)	Chernetidae	*Lamprochernes nodosus* (Schrank, 1803)^54^	BE	Leclercq (1945)
*Stomoxys calcitrans* (Linnaeus, 1758)	Chernetidae	*Lamprochernes savignyi* (Simon, 1881)^67^	IE	Kew (1916)
*Stomoxys calcitrans* (Linnaeus, 1758)	Chernetidae	*Lamprochernes savignyi* (Simon, 1881)^26^	GB-ENG	Jones (1978)
*Stomoxys* Geoffroy, 1762	Chernetidae	*Lamprochernes savignyi* (Simon, 1881)	ZA	Beier (1964)
*Stomoxys* Geoffroy, 1762	Chernetidae	*Pselaphochernes scorpioides* (Hermann, 1804)^68^	GB-ENG	Graham-Smith (1916)
** Neriidae **				
*Odontoloxozus longicornis* (Coquillett, 1904)	Chernetidae	*Dinocheirus arizonensis* (Banks, 1901)	US-AZ	Zeh and Zeh (1992b) ^69^
** Pantophthalmidae **				
*Pantophthalmus tabaninus* Thunberg, 1819	Chernetidae	*Semeiochernes armiger* (Balzan, 1892)	PA	Zeh and Zeh (1992a)
*Pantophthalmus tabaninus* Thunberg, 1819	Chernetidae	*Semeiochernes armiger* (Balzan, 1892)^70^	BR	Santos et al. (2005)
Not specified	Chernetidae	*Semeiochernes armiger* (Balzan, 1892)^71^	BR	Mahnert (1987)
** Sarcophagidae **				
Not specified	Chernetidae	*Anthrenochernes stellae* Lohmander, 1939	SE	Gärdenfors and Wilander (1995) ^72^
** Scatopsidae **				
*Scatopse notata* (Linnaeus, 1758)	Chernetidae	*Pselaphochernes scorpioides* (Hermann, 1804)	FR	Present study
** Scenopinidae **				
*Scenopinus fenestralis* (Linnaeus, 1758)^73^	Chernetidae	*Lamprochernes nodosus* (Schrank, 1803)	FR	Vachon (1940)
** Sciomyzidae **				
*Pherbellia annulipes* (Zetterstedt, 1846)	Chernetidae	*Pselaphochernes scorpioides* (Hermann, 1804)	SK	Present study
** Sphaeroceridae **				
*Limosina silvatica* (Meigen, 1830)^74^	Chernetidae	*Pselaphochernes scorpioides* (Hermann, 1804)	GB-ENG	Cuthbertson (1982)
Not specified	Chernetidae	*Pselaphochernes scorpioides* (Hermann, 1804)	Europe	Weygoldt (1966, 1969)
** Stratiomyidae **				
*Artemita amenides* (Walker, 1849)	Chernetidae	Parachernes (P.) setiger Mahnert, 1979	BR	Guimarães et al. (2025)
*Dicranophora bispinosa* (Wiedemann, 1830)^75^	Chernetidae	*Lamprochernes nodosus* (Schrank, 1803)*^75^	FR	Vachon (1940)
*Sargus bipunctatus* (Scopoli, 1763)	Chernetidae	*Pselaphochernes scorpioides* (Hermann, 1804)	DE	Ziegler (2004)
*Sargus iridatus* (Scopoli, 1763)^76^	Chernetidae	*Lamprochernes nodosus* (Schrank, 1803)	GB-ENG	Jones (1978)
*Sargus iridatus* (Scopoli, 1763)	Chernetidae	*Lamprochernes nodosus* (Schrank, 1803)	GB-ENG	Judson (1979)
** Syrphidae **				
*Brachyopa bicolor* (Fallén, 1817)	Chernetidae	*Chernes hahnii* (C.L. Koch, 1839)	AT	Červená et al. (2019)
*Brachypalpus laphriformis* (Fallén, 1816)	Chernetidae	*Lamprochernes nodosus* (Schrank, 1803)*^1^	DE	Gerstaecker (1862)
*Eristalis arbustorum* (Linnaeus, 1758)	Chernetidae	*Lamprochernes nodosus* (Schrank, 1803)	GB-ENG	Jones (1978)
*Myathropa florea* (Linnaeus, 1758)	Chernetidae	*Anthrenochernes stellae* Lohmander, 1939	DE	Ssymank and Muster (2010)
*Myolepta dubia* (Fabricius, 1805)	Chernetidae	*Pselaphochernes lacertosus* (L. Koch, 1873)	ES	Ricarte et al. (2016)
*Volucella zonaria* (Poda, 1761)	Chernetidae	*Lamprochernes nodosus* (Schrank, 1803)^77^	FR	Berland (1932); Vachon (1940)
Not specified^78^	Chernetidae	*Lamprochernes savignyi* (Simon, 1881)	ZA	Beier (1958)
** Tabanidae **				
*Phorcotabanus cinereus* (Wiedemann, 1821)	Chernetidae	Parachernes (P.) inpai Mahnert, 1979	BR	Aguiar and Bührnheim (1998b)
*Phorcotabanus cinereus* (Wiedemann, 1821)	Chernetidae	Parachernes (P.) melanopygus Beier, 1959	BR	Aguiar and Bührnheim (1998b)
*Phorcotabanus cinereus* (Wiedemann, 1821)	Chernetidae	Parachernes (P.) plumosus (With, 1908)	BR	Aguiar and Bührnheim (1998b)
*Stenotabanus cretatus* Fairchild, 1961	Chernetidae	Parachernes (P.) melanopygus Beier, 1959	BR	Aguiar and Bührnheim (1998b)
*Stenotabanus cretatus* Fairchild, 1961	Chernetidae	Parachernes (P.) plumosus (With, 1908)	BR	Aguiar and Bührnheim (1998b)
*Tabanus amapaensis* Fairchild 1961	Chernetidae	Parachernes (P.) melanopygus Beier, 1959	BR	Aguiar and Bührnheim (1998b)
*Tabanus occidentalis* Linnaeus, 1758	Chernetidae	Parachernes (P.) inpai Mahnert, 1979	BR	Aguiar and Bührnheim (1998b)
*Tabanus trivittatus* Fabricius, 1805	Chernetidae	Parachernes (P.) plumosus (With, 1908)	BR	Aguiar and Bührnheim (1998b)
*Tabanus* Linnaeus, 1758	Not specified	Not specified	AR	Berg (1893)
Not specified	Chernetidae	Parachernes (P.) argentatopunctatus (Ellingsen, 1910)	GY	Beier (1948)
** Tachinidae **				
*Exorista larvarum* (Linnaeus, 1758)^79^	Chernetidae	*Lamprochernes nodosus* (Schrank, 1803)^79^	GB-ENG	Anonymous (1834)
*Zelia vertebrata* (Say, 1829)	Not specified	Not specified	N America	Marshall (2012)
Not specified^80^	Chernetidae	*Hesperochernes pallipes* (Banks, 1893)^80^	US-CO	Banks (1895)
** Tanypezidae **				
*Neotanypeza* Hendel, 1903	Not specified	Not specified	CR	Marshall (2012)
** Tipulidae **				
*Ctenophora pectinicornis* (Linnaeus, 1758)	Chernetidae	*Anthrenochernes stellae* Lohmander, 1939	SE	Gärdenfors and Wilander (1995)
*Ctenophora pectinicornis* (Linnaeus, 1758)	Chernetidae	*Lamprochernes nodosus* (Schrank, 1803)^81^	DE	Wagner (1892); Berland (1932)
*Ctenophora pectinicornis* (Linnaeus, 1758)	Not specified	Not specified	FR	Vachon (1953)
*Dictenidia bimaculata* (Linnaeus, 1761)	Chernetidae	*Chernes cimicoides* (Fabricius, 1793)	GB-ENG	Bloxham and Smart (2001)
*Dictenidia bimaculata* (Linnaeus, 1761)	Chernetidae	*Chernes cimicoides* (Fabricius, 1793)	CZ	Christophoryová and Krásenský (2022)
*Tipula pabulina* Meigen, 1818	Chernetidae	*Allochernes peregrinus* Lohmander, 1939	CH	DeVore-Scribante (1999)
*Tipula unicincta* Doane, 1901	Syarinidae	*Syarinus obscurus* (Banks, 1893)	US	Muchmore (1971)
*Tipula vernalis* Meigen, 1804	Cheliferidae	*Dactylochelifer degeerii* (C.L. Koch, 1835)	FR	Present study
*Tipula* Linnaeus, 1758	Chernetidae	*Lamprochernes nodosus* (Schrank, 1803)^82^	GB-ENG	Jenyns (1846)
*Tipula* Linnaeus, 1758	Syarinidae	*Syarinus obscurus* (Banks, 1893)^83^	US	Hagen (1879); Muchmore (1971)
*Zelandotipula* Alexander, 1922	Chernetidae	Not specified	BR	Matthiesen and Hahn (1981)
Not specified	Cheliferidae	*Dactylochelifer degeerii* (C.L. Koch, 1835)^84^	AT	Ressl (1995)
Not specified	Chernetidae	*Hesperochernes molestus* Hoff, 1956	US-TX	Muchmore (1992)
** Ulidiidae **				
*Ceroxys urticae* (Linnaeus, 1758)	Chernetidae	*Lamprochernes cf. chyzeri* (Tömösváry, 1883)	CZ	Present study
*Physiphora alceae* (Preyssler, 1791)^85^	Chernetidae	*Lamprochernes muscivorus* Redikorzev, 1949	TM	Redikorzev (1949)
*Physiphora alceae* (Preyssler, 1791)^85^	Chernetidae	*Lamprochernes muscivorus* Redikorzev, 1949	UZ	Redikorzev (1949)
*Physiphora alceae* (Preyssler, 1791)^85^	Chernetidae	*Lamprochernes muscivorus* Redikorzev, 1949	UZ	Redikorzev (1949)
*Physiphora alceae* (Preyssler, 1791)^86^	Chernetidae	*Lamprochernes nodosus* (Schrank, 1803)^86^	HU	Loew (1845)
*Physiphora alceae* (Preyssler, 1791)^87^	Chernetidae	*Lamprochernes nodosus* (Schrank, 1803)^87^	AT	Schiner (1872)
*Physiphora alceae* (Preyssler, 1791)^88^	Chernetidae	*Lamprochernes nodosus* (Schrank, 1803)	AT	Ressl (1965)
*Physiphora alceae* (Preyssler, 1791)^88^	Chernetidae	*Lamprochernes nodosus* (Schrank, 1803)	AT	Ressl (1970)
*Physiphora alceae* (Preyssler, 1791)^88^	Chernetidae	*Lamprochernes nodosus* (Schrank, 1803)	GB-ENG	Clements (1987)
*Physiphora alceae* (Preyssler, 1791)^88^	Chernetidae	*Lamprochernes nodosus* (Schrank, 1803)	RO	Georgescu and Capuse (1996)
*Physiphora alceae* (Preyssler, 1791)	Chernetidae	*Lamprochernes nodosus* (Schrank, 1803)	SK	Christophoryová et al. (2018)
*Physiphora alceae* (Preyssler, 1791)	Chernetidae	*Lamprochernes nodosus* (Schrank, 1803)	SK	Christophoryová et al. (2018)
*Physiphora alceae* (Preyssler, 1791)	Chernetidae	*Lamprochernes nodosus* (Schrank, 1803)	SK	Červená et al. (2019)
*Physiphora alceae* (Preyssler, 1791)	Not specified	Not specified^89^	FR	Livory (2021)
*Physiphora clausa* Macquart, 1843^90^	Chernetidae	*Lamprochernes savignyi* (Simon, 1881)	MU	Beier (1953)
*Physiphora* Fallén, 1810	Chernetidae	*Lamprochernes nodosus* (Schrank, 1803)	FR	Present study
*Ulidia erythrophthalma* Meigen, 1826	Chernetidae	*Lamprochernes nodosus* (Schrank, 1803)^91^	AT	Löw (1866)
** Xylophagidae **				
*Xylophagus ater* Meigen, 1804	Chernetidae	*Chernes cimicoides* (Fabricius, 1793)	SK	Present study
** Diptera **				
Not specified	Cheliferidae	*Chelifer cancroides* (Linnaeus, 1758)	FR	Moniez (1893)
Not specified	Cheliferidae	*Chelifer loewi* Hagen, 1879^92^	PA	Hagen (1879)
Not specified	Chernetidae	*Chernes sanborni* Hagen, 1868^93^	N America	Hagen (1867, 1868); Muchmore (1971)
Not specified	Chernetidae	*Chernes sanborni* Hagen, 1868^94^	US-MA	Hagen (1879)
Not specified	Chthoniidae	*Ephippiochthonius tetrachelatus* (Preyssler, 1790)	HU	Novák (2024)
Not specified	Chernetidae	*Hesperochernes* Chamberlin, 1924	US	Muchmore (1971) ^95^
Not specified	Chernetidae	*Lamprochernes chyzeri* (Tömösváry, 1883)	GB-ENG	Jones (1978)
Not specified	Chernetidae	*Lamprochernes chyzeri* (Tömösváry, 1883)	AT	Ressl (1995)
Not specified	Chernetidae	*Lamprochernes chyzeri* (Tömösváry, 1883)	CH	DeVore-Scribante (1999)
Not specified	Chernetidae	*Lamprochernes chyzeri* (Tömösváry, 1883), *Lamprochernes nodosus* (Schrank, 1803)	DE	Drogla and Lippold (2004)
Not specified	Chernetidae	*Lamprochernes nodosus* (Schrank, 1803)^82^	GB-ENG	Jenyns (1846)
Not specified	Chernetidae	*Lamprochernes nodosus* (Schrank, 1803)*^1^	AT	Löw (1867)
Not specified	Chernetidae	*Lamprochernes nodosus* (Schrank, 1803)^54^	FR	Moniez (1893)
Not specified^96^	Chernetidae	*Lamprochernes nodosus* (Schrank, 1803)^96^	GB-ENG	Kew (1901) ^96^
Not specified	Chernetidae	*Lamprochernes nodosus* (Schrank, 1803)^47^	GB-ENG	Kew (1904)
Not specified	Chernetidae	*Lamprochernes nodosus* (Schrank, 1803)^47^	GB-SCT	Godfrey (1907, 1908)
Not specified	Chernetidae	*Lamprochernes nodosus* (Schrank, 1803)	SE	Tullgren (1911); Lohmander (1939a)
Not specified	Chernetidae	*Lamprochernes nodosus* (Schrank, 1803)	ES	Navás (1918)
Not specified	Chernetidae	*Lamprochernes nodosus* (Schrank, 1803)^47^	ZA	Godfrey (1921)
Not specified	Chernetidae	*Lamprochernes nodosus* (Schrank, 1803)	FR	Vachon (1954)
Not specified	Chernetidae	*Lamprochernes nodosus* (Schrank, 1803)	DE	Zielke (1969)
Not specified	Chernetidae	*Lamprochernes nodosus* (Schrank, 1803)	DE	Zielke (1969)
Not specified	Chernetidae	*Lamprochernes nodosus* (Schrank, 1803)	DE	Zielke (1969)
Not specified	Chernetidae	*Lamprochernes nodosus* (Schrank, 1803)	GB-ENG	Jones (1978)
Not specified^97^	Chernetidae	*Lamprochernes nodosus* (Schrank, 1803)	GB-ENG	Judson (1987)
Not specified	Chernetidae	*Lamprochernes nodosus* (Schrank, 1803)	CH	DeVore-Scribante (1999)
Not specified	Chernetidae	*Lamprochernes nodosus* (Schrank, 1803)	DE	Drogla and Lippold (2004)
Not specified	Chernetidae	*Lamprochernes nodosus* (Schrank, 1803)	IR	Nassirkhani and Shoushtari (2016)
Not specified	Chernetidae	*Lamprochernes savignyi* (Simon, 1881)^98^	GB-ENG	Ellingsen (1907)
Not specified	Chernetidae	*Lamprochernes savignyi* (Simon, 1881)^98^	GB-ENG	Ellingsen (1907)
Not specified	Chernetidae	*Lamprochernes savignyi* (Simon, 1881)^99^	GB-ENG	Kew (1911) ^99^
Not specified	Chernetidae	*Lamprochernes savignyi* (Simon, 1881)^58^	IE ^58^	Kew (1916)
Not specified	Chernetidae	*Lamprochernes savignyi* (Simon, 1881)	SD	Beier (1946); Beier (1948)^100^
Not specified	Chernetidae	*Lamprochernes savignyi* (Simon, 1881)	SD	Beier (1953)
Not specified	Chernetidae	*Lamprochernes savignyi* (Simon, 1881)	ZA	Beier (1958)
Not specified	Chernetidae	*Lamprochernes savignyi* (Simon, 1881)	IL	Beier (1963)
Not specified	Chernetidae	*Lamprochernes savignyi* (Simon, 1881)	ZA	Beier (1964)
Not specified	Chernetidae	*Lamprochernes savignyi* (Simon, 1881)	NZ	Beier (1969)
Not specified	Chernetidae	*Lamprochernes savignyi* (Simon, 1881)^26^	GB-ENG	Judson (1979)
Not specified	Chernetidae	*Lamprochernes savignyi* (Simon, 1881)	IE	Harvey (1987)
Not specified	Chernetidae	*Lamprochernes savignyi* (Simon, 1881)	IL	Warburg et al. (2023a)
Not specified	Chernetidae	Parachernes (P.) melanopygus Beier, 1959	BR	Aguiar and Bührnheim (1998b)
Not specified	Chernetidae	*Phymatochernes crassimanus* Mahnert, 1979	BR	Aguiar and Bührnheim (1998b)
Not specified	Chernetidae	*Pselaphochernes scorpioides* (Hermann, 1804)^101^	GB-ENG	Kew (1929)
Not specified	Chernetidae	*Pselaphochernes scorpioides* (Hermann, 1804)	DE	Drogla and Lippold (2004)
Not specified	Not specified	Not specified^63^	US	Knab (1897); Muchmore (1971)
Not specified	Not specified	Not specified	NZ	Edwards (1955)

Numbers in superscript refer to the *Notes*, and the asterisk (*) indicates the interpretation of Beier (1948), see Results. Abbreviations: AR – Argentina; AT – Austria; AU – Australia; BE – Belgium; BR – Brazil; CH – Switzerland; CR – Costa Rica; CZ – Czech Republic; DE – Germany; DK – Denmark; EC – Ecuador; ES – Spain; FI – Finland; FR – France; GB-ENG – United Kingdom, England; GB-SCT – United Kingdom, Scotland; GH – Ghana; GY – Guyana; HU – Hungary; IE – Ireland; IL – Israel; IR – Iran; IT – Italy; JP – Japan; KG – Kyrgyzstan; MU – Mauritius; N America – North America; NL – Netherlands; NZ – New Zealand; PA – Panama; RO – Romania; SD – Sudan; SE – Sweden; SI – Slovenia; SK – Slovakia; TM – Turkmenistan; TN – Tunisia; US – United States of America; US-AZ – United States of America, Arizona; US-CA – United States of America, California; US-CO – United States of America, Colorado; US-MA – United States of America, Massachusetts; US-PA – United States of America, Pennsylvania; US-TX – United States of America, Texas; UZ – Uzbekistan; ZA – South Africa.

**Figure 2.**
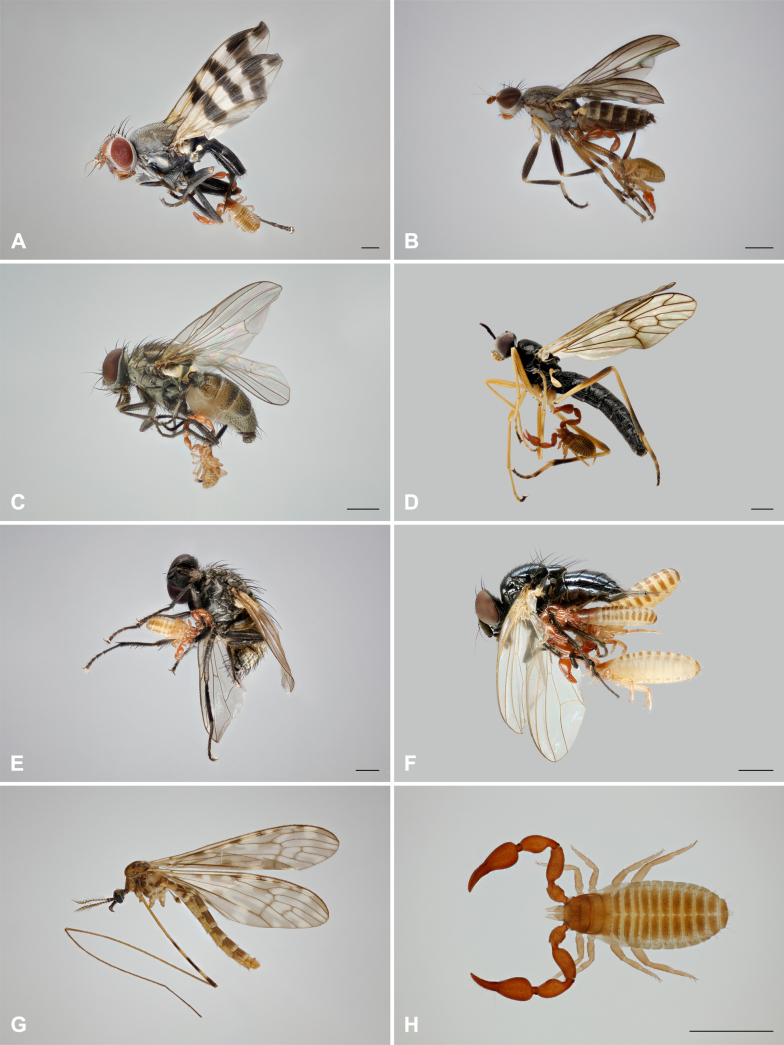
Cases of phoresy from Czechia, Slovakia, and Slovenia. **A**. *Ceroxys urticae* and *Lamprochernes cf. chyzeri* (locality 1); **B**. *Pherbellia annulipes* and *Pselaphochernes scorpioides* (locality 6); **C**. *Fannia canicularis* and *P. scorpioides* (locality 7); **D**. *Xylophagus ater* and *Chernes cimicoides* (locality 8); **E**. *Musca autumnalis* and *L. cf. chyzeri* (locality 9); **F**. *Lonchaea chorea* and *L. cf. chyzeri* (locality 10); **G**, **H**. *Limonia nubeculosa* and *P. scorpioides* (locality 11). Scale bars: 1 mm.

**Figure 3.**
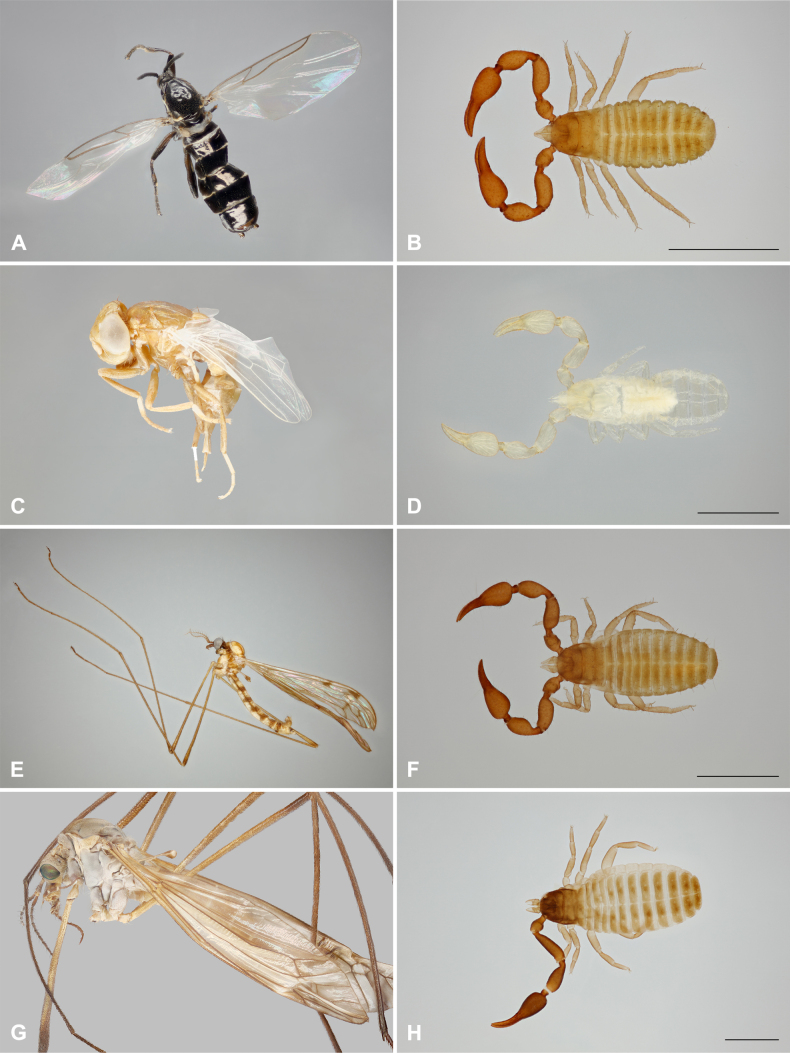
Cases of phoresy from France. **A**, **B**. *Scatopse notata* and *Pselaphochernes scorpioides* (locality 2); **C**, **D**. *Physiphora* sp. and *Lamprochernes nodosus* (locality 3); **E**, **F**. *Neolimonia dumetorum* and *P. scorpioides* (locality 4); **G**, **H**. *Tipula vernalis* and *Dactylochelifer degeerii* (locality 5). Scale bars: 1 mm.

1. Czechia • 1 ♀ of *Lamprochernes cf. chyzeri* attached to femur III of 1 ♀ of *Ceroxys urticae* (Ulidiidae); Lysá nad Labem, Hrabanovská černava National Nature Monument; 50.2188°N, 14.8358°E; 186 m a.s.l.; 13 Jun.–8 Jul. 2024; Jindřich Roháček leg.; wetland, lowland fen, Malaise trap; host Jindřich Roháček det., deposited in the Silesian Museum, Opava, Czechia; FO 40/6 (Fig. 2A).

2. France • 1 ♀ of *Pselaphochernes scorpioides* attached to the base of femur III of 1 ♂ of *Scatopse notata* (Scatopsidae); Bénouville; 49.2444, -0.2865; 23 m a.s.l.; 15 Oct. 2023; Vincent Mathy leg.; family house garden, compost bin walls, individual sampling; host Vincent Mathy det.; FO 41/6 (Fig. 3A, B).

3. France • 1 ♀ of *Lamprochernes nodosus* phoretic on the leg of 1 ♀ of *Physiphora* sp. (Ulidiidae); Mauges-sur-Loire; 47.3311, -1.0361; 56 m a.s.l.; 29 Aug. 2016; Olivier Gabory leg.; inside a house, individual sampling; host Jindřich Roháček det.; FO 42/6 (Fig. 3C, D).

4. France • 1 ♀ of *P. scorpioides* attached to trochanter I of 1 ♂ of *Neolimonia dumetorum* (Limoniidae); Vernet-les-Bains; 42.5437°N, 2.3842°E; 690 m a.s.l.; 22 May 2023; Julien Tchilinguirian leg.; pine forest, camping ground, individual sampling; host Levente-Péter Kolcsár det.; FO 43/6 (Fig. 3E, F).

5. France • 1 ♀ of *Dactylochelifer degeerii* phoretic on the leg of 1 ♀ of *Tipula vernalis* (Tipulidae); Vimont; 49.1490, -0.1709; 5 m a.s.l.; 9 May 2021; Antoine Racine leg.; swamp area, individual sampling; host Antoine Racine det.; FO 44/6 (Fig. 3G, H).

6. Slovakia • 1 ♀ of *P. scorpioides* attached between trochanter and femur III of 1 ♀ of *Pherbellia annulipes* (Sciomyzidae); Bratislava, Botanical Garden; 48.1465°N, 17.0753°E; 144 m a.s.l.; 3–12 Jul. 2024; Simona Chamulová, Jana Christophoryová leg.; botanical garden, Malaise trap; host Jindřich Roháček det.; FO 45/6 (Fig. 2B).

7. Slovakia • 1 ♀ of *P. scorpioides* attached to the base of femur III of 1 ♂ of *Fannia canicularis* (Fanniidae); Bratislava, Police Academy; 48.2030°N, 17.1720°E; 136 m a.s.l.; 17 Apr. 2024; Lucia Švecová leg.; grassy area, pyramid trap; host Miroslav Barták det.; FO 46/6 (Fig. 2C).

8. Slovakia • 1 ♀ of *Chernes cimicoides* attached between trochanter and femur III of 1 ♂ of *Xylophagus ater* (Xylophagidae); Cerová vrchovina Highlands, Fenek Protected Area; 48.1847°N, 20.0416°E; 240 m a.s.l.; 3–25 May 2022; Jindřich Roháček, Jan Ševčík, Michal Tkoč leg.; alder forest, Malaise trap; host Jindřich Roháček det., deposited in the Silesian Museum, Opava, Czechia; FO 47/6 (Fig. 2D).

9. Slovakia • 1 ♂ of *L. cf. chyzeri* attached between trochanter and femur II of 1 ♂ of *Musca autumnalis* (Muscidae); Ivanka pri Dunaji, Bažantnica; 48.1942°N, 17.2675°E; 130 m a.s.l.; 12 May 2022; Ján Samay, Ľubomír Vidlička leg.; overgrown meadow among trees, Malaise trap; host Erikas Lutovinovas det.; FO 48/6 (Fig. 2E).

10. Slovakia • 2 ♀♀, 1 ♂ of *L. cf. chyzeri* attached to femora III of 1 ♀ of *Lonchaea chorea* (Lonchaeidae); Kežmarok, Čajka Ranch; 49.1587°N, 20.4528°E; 630 m a.s.l.; 3–5 Aug. 2022; Ema Hetešová leg.; ranch area, Malaise trap; host Ian MacGowan det.; FO 49/6 (Fig. 2F).

11. Slovenia • 1 ♀ of *P. scorpioides* attached to the leg of 1 ♂ of *Limonia nubeculosa* (Limoniidae); Otoška Jama, Veliki Otok; 45.7918°N, 14.1970°E; 543 m a.s.l.; 1 Aug. 2024; Pavol Purgat, Lucia Švecová leg.; cave entrance, individual sampling; host Levente-Péter Kolcsár det.; FO 50/6 (Fig. 2G, H).

### Revision of published data on phoresy from Slovakia (Table 1)

- Christophoryová et al. (2017a): In *Allochernes peregrinus*, the host had originally been reported only as Diptera. In the present study, a female of *Hilara* (Empididae; det. Miroslav Barták) was identified based on photographic documentation. The specimen is deposited in the collection of Dr Viera Stloukalová, but could not be located. In addition, three further phoretic records had originally been identified only to the genus *Lamprochernes* with their hosts recorded merely as Diptera. In the July sample, the host was identified as a female of *Stomoxys calcitrans* (Muscidae; det. Miroslav Barták), and the phoront as *Lamprochernes cf. chyzeri* (det. J. Christophoryová). In the August sample, the pseudoscorpions were identified as *L. nodosus* (det. J. Christophoryová), with the host identified as a male of *Musca domestica* (Muscidae; det. Miroslav Barták).
- Christophoryová et al. (2018): The host originally identified only to the superfamily Muscoidea was reidentified as *Delia platura* (Anthomyiidae; det. Miroslav Barták, Samuel Krčmárik).
- Červená et al. (2019): The host previously assigned to Empididae was reidentified as a female of *Leptopeza flavipes* (Hybotidae; det. Miroslav Barták, Jindřich Roháček).
- Christophoryová et al. (2021): The host was lost during examination by a specialist and could therefore be identified only to the genus level based on photographic documentation.
- Hetešová and Christophoryová (2022): The host belonging to Chyromyidae was identified as a female of *Chyromya flava* (det. Jindřich Roháček).

### Published data on the phoresy of Pseudoscorpiones and Diptera worldwide

Altogether, 172 published records as well as new records (marked as “Present study”) documenting phoretic associations between pseudoscorpions and dipterans, spanning the period from 1761 to 2025, were compiled and evaluated (Table 1).

The present study expands the original dataset of Poinar et al. (1998) by incorporating 100 additional relevant publications published up to and including the year 1998 (Adams 1787; Hermann 1804; Leach 1817; Kirby and Spence 1826; Anonymous 1831, 1832, 1834, 1875, 1879; Ed 1834; Lukis 1834; Moore 1834; Loew 1845; Jenyns 1846; Clarke 1858; Stainton 1865; Stevens 1866; Hagen 1867, 1868; Löw 1867; M’lntire 1869; Bisshopp 1871; Koch 1873; Newman 1875; Stecker 1875; Leidy 1877; Dale 1878; Simon 1879; Courtois de Langlade 1883; Pickard-Cambridge 1885, 1892; Mégnin 1886; Moniez 1893; Hess 1894; Müller and Schenkel 1895; Knab 1897; Kew 1901, 1904, 1911, 1916, 1929; Fenizia 1902; Hickson 1905; Hill 1905; Pocock 1905; Frey-Gessner 1906; Ellingsen 1907, 1908, 1913; Godfrey 1907, 1908, 1909, 1921; Jackson 1907, 1908; Tullgren 1907, 1911; Hewitt 1910, 1914; Stephens 1910; Lessert 1911; Carpenter 1912; Franganillo 1913; Navás 1918, 1925; Grimpe 1921; Caporiacco 1923; Beier 1929, 1939, 1946, 1951, 1953, 1958, 1963, 1964, 1969; Vachon 1934a, 1934b, 1953, 1954; Lohmander 1939a; Leclercq 1945; Kaisila 1949; Redikorzev 1949; Hinton 1954; Edwards 1955; Ressl and Beier 1958; Meinertz 1964; Ressl 1965, 1970, 1995; Benedict and Malcolm 1977; Judson 1979, 1987; Meyer et al. 1985; Andersen 1987; Harvey 1987; Muchmore 1992; Georgescu and Capuse 1996; Bellmann 1997).

In subsequent years, additional data on phoresy became available, and records published from 1998 onwards were compiled in the present study, resulting in the inclusion of 31 further publications (Aguiar and Bührnheim 1998b; DeVore-Scribante 1999; Bloxham and Smart 2001; Castillo-Miralbes 2002; Drogla and Lippold 2004; Ziegler 2004; Santos et al. 2005; Szymkowiak et al. 2007; Domínguez et al. 2008; Ssymank and Muster 2010; Christophoryová et al. 2011, 2017a, 2018, 2021; Marshall 2012; Lira et al. 2014; Nassirkhani and Shoushtari 2016; Ricarte et al. 2016; Lira and Tizo-Pedroso 2017; Mederos and Zaragoza 2017; Zaragoza and Ruiz de la Cuesta Santiago 2017; Červená et al. 2019; Martínez et al. 2020; Livory 2021; Christophoryová and Krásenský 2022; Hetešová and Christophoryová 2022; Warburg et al. 2023a; Jong et al. 2024; Novák 2024; Christophoryová and Blažej 2025; Guimarães et al. 2025).

Records marked with an asterisk (*) in Table 1 indicate cases in which host or phoront identifications follow the interpretation of Beier (1948), representing the most recent traceable taxonomic treatment of the originally cited material; these identifications have not been confirmed in WPC (2026). Missing data from published records are marked as “Not specified” in Table 1. Table 1 includes numbered *Notes* (1–101) documenting revisions and corrections to published records, including changes in host and phoront names, updates to family assignments, spelling errors in original sources (marked as [sic]), additions of data based on photographic documentation, correction of erroneous information, adjustments of country records, identification of duplicate data, and related amendments.

Hosts illustrated in Bellmann (1997) and Ziegler (2004) were identified from photographs as *Musca domestica* and *Sargus bipunctatus*, respectively, by a dipteran specialist (det. Alexssandro Camargo). The host from Szymkowiak et al. (2007) was identified only on the family level as Micropezidae (det. Nathalie Fial, Nikolaus Szucsich).

### Reassessment of records summarised by Poinar et al. (1998)

The dataset compiled by Poinar et al. (1998), representing the most comprehensive synthesis of pseudoscorpion–dipteran phoresy at the time, was critically re-evaluated in the present study. Although several of the following publications were included in Poinar et al. (1998), not all available data on phoresy were extracted, and additional records were identified through re-examination of the original publications:

- Hagen (1879): records of *Syarinus obscurus* (reported as *Obisium*) associated with *Tipula*; and *Chernes sanborni* associated with Diptera.
- Graham-Smith (1916): records of *Lamprochernes nodosus* associated with *Musca domestica*.
- Burr (1919): record of *L. nodosus* associated with *M. domestica*.
- Berland (1932): records of *L. nodosus* associated with *M. domestica* and *Fannia canicularis*.
- Vachon (1940): records of *L. nodosus* associated with *Stomoxys calcitrans*.
- Muchmore (1971): Two records of *Hesperochernes* associated with Diptera.
- Jones (1978): records of *Chelifer cancroides* associated with *M. domestica*; *Lamprochernes chyzeri* associated with Diptera; and *L. nodosus* associated with *F. canicularis*, *M. domestica*, *Lonchaea*, and Diptera.
- Mahnert (1987): record of *Parachernes beieri* associated with *Drosophila aff. repleta*.
- Addendum in Gärdenfors and Wilander (1995): record of *Anthrenochernes stellae* associated with Sarcophagidae.

The following records compiled in Poinar et al. (1998) were found to contain inaccuracies when re-examined against the original sources:

- Data on *Lamprochernes nodosus* phoretic on *Stomoxys calcitrans* were attributed to Lukis (1831) and Schiner (1872). However, Lukis (1831) represents only a response to correspondence from Anonymous (1831) and provides no original data. Moreover, Schiner (1872) reported the host only as *Chloria demandata* (currently *Physiphora alceae*). The host *S. calcitrans* listed in Poinar et al. (1998) is therefore not supported by the original sources.
- Records from Zielke (1969) and Macrae (1869) were cited as *L. nodosus* on *Fannia canicularis*. The record from Macrae (1869) is supported, whereas Zielke (1969) indicates different associations (see Table 1).
- The phoront in Webster (1897) was listed as *Chernes sanborni* on *Musca domestica*. However, according to Muchmore (1971), cited therein, this identification is not supported; the phoront is therefore treated as not specified.
- The record of *Lamprochernes savignyi* on Tabanidae is attributed to Ellingsen (1913); however, this association could not be verified in the original paper.
- The record attributed to Vachon (1954) for *L. nodosus* on *Drosophila funebris* is not supported by the original source (here treated as Vachon 1954), in which this host association is not reported.
- Data on *L. savignyi* from New Zealand, reported as phoretic on unidentified Tipulidae and attributed to Muchmore (1971), could not be verified in the original source.
- Data on the phoresy of *L. nodosus* on Diptera attributed to Redikorzev (1936) are imprecise. The original publication contains only an illustration depicting *L. nodosus* phoretic on *M. domestica* from the former USSR and does not provide supporting records from Asia.
- *Lamprochernes nodosus* on Diptera from the Pacific was cited based on Guilmette Jr et al. (1970). However, the original material was obtained using a suction trap, and no host taxon was specified; consequently, this record was not considered evidence of phoresy on Diptera in the present study.
- The phoresy of *Chelifer cancroides* on *M. domestica* in Europe was attributed to Nonidez (1917). However, this does not represent original data from Nonidez (1917) but a citation from Franganillo (1913).
- The record from Donovan (1797) appears twice, under *C. cancroides* and *L. nodosus*, which is most likely an error (see Note 7 for details).
- The host family cited from Löw (1866) and Clements (1987) is given as Otitidae; this family is currently merged with Ulidiidae.
- The host cited from Macrae (1869) is misspelled as *Fannia canalicularis* [sic] (should be *Fannia canicularis*), and the family is given as Muscidae, whereas it is currently treated as Fanniidae.
- The host *Sargus iridatus* cited from Jones (1978) is placed in Syrphidae, whereas it correctly belongs to Stratiomyidae.
- The host cited from Jones (1978) is given as the subgenus *Achyrolimonia* within the genus *Limonia*; it is currently treated as the genus *Achyrolimonia*.
- The host *Hydrotaea ignava* cited from Graham-Smith (1916) is listed under its former name and family (see Note 27 for details).
- The pseudoscorpion cited from Hagen (1879) is given as *Lamprochernes loewi* (Hagen, 1879), but this name should be treated as *Chelifer loewi*, a nomen nudum.
- The host cited from Burr (1919) is given as *Tephrochlamis* [sic] canescens (should be *Tephrochlamys canescens* (Meigen, 1830)).
- The host genus cited from Zeh and Zeh (1992c) as *Odontolorozuz* [sic] should be corrected to *Odontoloxozus* Enderlein, 1922 (treated here as Zeh and Zeh 1992b).
- The pseudoscorpion cited from Jones (1978) as *Lamprochernes scorpioides* represents an incorrect name combination and should be *L. chyzeri*.

The first comprehensive synthesis of published data on pseudoscorpion phoresy was provided by Beier (1948), who compiled numerous published records and added only three new observations (see Table 1). Poinar et al. (1998) aimed to include these new data in their synthesis; however, re-examination shows that some records treated as new in Poinar et al. (1998) are based on secondary citations of earlier publications or require correction. These cases are outlined below:

- The record of *Lustrochernes argentinus* (Thorell, 1877) on *Lucilia* Robineau-Desvoidy, 1830 from South America, attributed to Beier (1948), appears to be based on a misinterpretation of the original source. In Beier (1948: 452), the host is *Lucilius* Kuwert, 1891 (= *Passalus* Fabricius, 1792; Coleoptera).
- Data on *Allochernes wideri* (currently *Lamprochernes nodosus*) phoretic on *Ulidia erythrophthalma* were treated as new data from Beier (1948). However, in Beier (1948), this record is cited from Löw (1866) and therefore does not represent a new observation.
- Data on *Syarinus obscurus* phoretic on *Tipula* from North America were treated as new data from Beier (1948). However, in Beier (1948), this record is cited from Hagen (1879), with additional data later provided by Muchmore (1971).
- Records attributed to Beier (1948) list *L. nodosus* and *Chelifer cancroides* phoretic on *Musca meteorica* (currently *Hydrotaea meteorica*) and on *Tachina larvarum* (currently *Exorista larvarum*). According to the WPC (2026), these records should be referred exclusively to *L. nodosus*, as *C. cancroides* represents a misidentification. Furthermore, these data are not original to Beier (1948) but were cited therein from Anonymous (1834).
- Data from Beier (1948) on *L. savignyi* from Africa (Egypt, Sudan) were supplemented with the host *Eutrichota apicalis* (reported as *Pegomya apicalis*). However, in the original source (Beier 1948), the host is mentioned only as Diptera. In addition, Europe is listed as a region for this phoretic association, although the original publication does not support this.
- Data on *L. nodosus* phoretic on *M. domestica* from Austria reported by Beier (1948) were not included.
- Two species (*L. nodosus* and *Chernes cimicoides*) are mentioned with the host *Ctenophora pectinicornis*, attributed to Wagner (1892). However, only data referring to *L. nodosus* should be considered (see Note 81 for details).

### Notes

^1^ Pseudoscorpion as *Chelifer* Geoffroy, 1762; in Beier (1948), as *Lamprochernes nodosus*.

^2^ Host as *Hylemyia floralis* (Fallén, 1824).

^3^ Host as *Pegomya* [sic] affinis Stein, 1898 (should be *Pegomyia affinis*).

^4^ Host as *Pegomya* [sic] apicalis (Stein, 1898) (should be *Pegomyia apicalis*).

^5^ Originally as “Schmeißfliege” and “Bücherskorpion” = Calliphoridae and *Chelifer cancroides*. In Beier (1948), the host is specified as *Calliphora erythrocephala* (Meigen, 1826), and the pseudoscorpion is redetermined as *Lamprochernes nodosus*.

^6^ Host originally reported as flies; one specimen from Mr Campbell was caught on the leg of a blow-fly, on a window in October 1887 (Kew 1901); the host was later specified as *Calliphora erythrocephala* (Beier 1948). Pseudoscorpion as *Chernes nodosus* Schrank, 1803.

^7^ Host as *Musca vomitoria* Linnaeus, 1758; pseudoscorpion as *Phalangium crancroides* [sic] (should be *Phalangium cancroides* (Linnaeus, 1758)). In Beier (1948), the record is listed as *L. nodosus* and *Chelifer cancroides*, with hosts given as *Musca domestica* and *M. vomitoria*. Due to these inconsistencies, only the original combination (*C. cancroides* on *M. vomitoria*) was used.

^8^ Host originally as *M. vomitoria* and pseudoscorpion as *C. cancroides*. In Beier (1948), the host is specified as *Calliphora vomitoria*, and the pseudoscorpion is redetermined as *L. nodosus*.

^9^ Host only as Calliphoridae; specified as *Calliphora* in Beier (1948).

^10^ Genus of the host as *Lucia* [sic] (should be *Lucilia*).

^11^ Host family as Rhinophoridae, currently placed in Calliphoridae.

^12^ Genus of the host as *Aëdes* (now *Aedes*).

^13^ Host as *Dolichopus acutirostris* [sic] (should be *Dolichopus acuticornis*); pseudoscorpion as *Microcreagris strandi* (Ellingsen, 1901). The attachment to the fly may have occurred in the net, as the specimen was presumably collected using a hand net.

^14^ Pseudoscorpion as Parachernes (Argentochernes) nigrimanus Beier, 1948; junior homonym, replaced by Parachernes (P.) beieri.

^15^ Host as fly; pseudoscorpion as *Chelifer latreillii* Leach, 1817. In Beier (1948), the host is specified as *Fannia canalicularis* [sic] (should be *Fannia canicularis*), and the pseudoscorpion is redetermined as *Lamprochernes nodosus*.

^16^ Host as *Homalomyia canicularis* (Linnaeus, 1761). Pseudoscorpion originally as *C. cancroides*; in Beier (1948) as *L. nodosus*.

^17^ Host as *Homalomyia canicularis*; pseudoscorpion as Chelifer (Chernes) nodosus Schrank, 1803.

^18^ Pseudoscorpion as *Pseudozaona* Beier, 1932; junior synonym of *Chelodamus* Chamberlin, 1925, synonymised by Muchmore (1984).

^19^ Host as *Amoebalaria* [sic] (should be *Amoebaleria*).

^20^ Host as *Heteromyza flavipes* Zetterstedt, 1838; in Beier (1948) as *Tephrochlamis* [sic] canescens (should be *Tephrochlamys canescens* (Meigen, 1830)). Pseudoscorpion as *Chernes* Menge, 1855; in Beier (1948) as *Lamprochernes nodosus* (Schrank, 1803).

^21^ Host as *Dicranomyia decemmaculata* (Loew, 1873).

^22^ Host as subgenus *Achyrolimonia* within the genus *Limonia*.

^23^ Pseudoscorpion as *Dactylochelifer latreillii latreillii* (Leach, 1817).

^24^ Host as *Lonchaea vaginalis* Fallén, 1820. Pseudoscorpion as *C. cancroides*; in Beier (1948) and WPC (2026) as *L. nodosus*.

^25^ Host as *L. vaginalis*. Pseudoscorpion as *Chernes nodosus*.

^26^ Pseudoscorpion as *Lamprochernes godfreyi* (Kew, 1911). Chelifer (Chernes) godfreyi Kew, 1911 is a junior synonym of *Lamprochernes savignyi* Simon, 1881, synonymised by Muchmore (1975).

^27^ Host changed from *Ophyra leucostoma* (Wiedemann, 1817) (Anthomyiidae) to *Hydrotaea ignava* (Harris, 1780) (Muscidae). Pseudoscorpion as *Chernes nodosus*.

^28^ Host as *Musca meteorica* Linnaeus, 1758. Pseudoscorpion as *C. cancroides* in the original paper; treated as a misidentification in WPC (2026).

^29^ Host as *Musca corvina* Fabricius, 1781; pseudoscorpion as *Chernes nodosus*.

^30^ Host only as a fly; specified as *M. domestica* in Beier (1948).

^31^ Pseudoscorpion as *Chelifer panzeri* Koch, 1836.

^32^ Pseudoscorpion as *Toxochernes panzeri* (C.L. Koch, 1836).

^33^ Host as a fly; pseudoscorpion as *Acarus cancroides* Linnaeus, 1758. This record is treated as a misidentification and referred to *L. nodosus* (Beier 1948; WPC 2026); the host is specified as *M. domestica* in Beier (1948).

^34^ Host as a fly; pseudoscorpion without precise identification. In Beier (1948), treated as *L. nodosus* on *M. domestica*.

^35^ Host as fly; in Beier (1948) treated as *M. domestica*. Pseudoscorpion as *Chelifer parasita* Hermann, 1804; a junior synonym of *L. nodosus*, synonymised by Simon (1879).

^36^ Host as flies; pseudoscorpion not mentioned in the original paper. In Beier (1948), treated as *L. nodosus* on *M. domestica*.

^37^ Host as *Musca carnaria* Linnaeus, 1758 (Anonymous 1831); pseudoscorpion not specified. Corrected to *M. domestica* and *C. cancroides* (as *Chélifer cancröìdes*) (Anonymous 1832). In Beier (1948), the phoront is treated as *L. nodosus*.

^38^ Pseudoscorpion as *C. cancroides* in the original paper; treated as a misidentification in Beier (1948) and WPC (2026).

^39^ Pseudoscorpion as Chelifers in the original paper; in Beier (1948) as *L. nodosus*.

^40^ Pseudoscorpion as Chelifers in the original paper. This record is treated as a misidentification and referred to *L. nodosus* (Beier 1948; WPC 2026).

^41^ Pseudoscorpion as *C. cancroides*; in Beier (1948), as *L. nodosus*.

^42^ Pseudoscorpion as *Chelifer reussii* C.L. Koch, 1843; a junior synonym of *L. nodosus*, synonymised by Simon (1879).

^43^ Host as a fly; pseudoscorpion as *C. cancroides*. In Beier (1948), referred to *L. nodosus* on *M. domestica*; the pseudoscorpion identification is confirmed in WPC (2026).

^44^ In the original paper, the host is mentioned only as flies; in Beier (1948), it is specified as *M. domestica*. Pseudoscorpion as *Obisium muscorum* Leach, 1817, treated as a misidentification and referred to *L. nodosus* (Beier 1948; WPC 2026).

^45^ Host as flies; pseudoscorpion as *Chelifer nodosus* Schrank, 1803. In Beier (1948), the host is specified as *M. domestica*.

^46^ Host as flies. Pseudoscorpion not specified in the original paper (Courtois de Langlade 1883); later cited, with the phoront given as *C. cancroides* in Mégnin (1886). This record is treated as a misidentification and referred to *L. nodosus* (Beier 1948; WPC 2026). Host as *M. domestica* in Beier (1948).

^47^ Pseudoscorpion as *Chernes nodosus* Schrank, 1803.

^48^ Host originally reported as flies; pseudoscorpion as *Chernes nodosus*. In Beier (1948), several fly records were specified as *M. domestica* (see Note 6).

^49^ Pseudoscorpion as *Chelifer nodosus*. The specimens cited in this work belong only partly to *L. nodosus* (three specimens), whereas one specimen belongs to *L. chyzeri*; for details, see Lessert (1911).

^50^ Pseudoscorpion in the original paper as Pseudoscorpiones (*Chelifer*?), as *L. nodosus* in Beier (1948).

^51^ Pseudoscorpion originally as *Chelifer cancroides* (Linnaeus, 1758), but Lessert (1911) suggested that it should be referred to Chelifer (Lamprochernes) nodosus Schrank, 1803.

^52^ Pseudoscorpion as Chelifer (Lamprochernes) nodosus Schrank, 1803.

^53^ The pseudoscorpion is given as *Chernes nodosus* on flies. Beier (1948) redetermined the host as *M. domestica* but incorrectly cited Kew (1916). The record in Kew (1916) is itself cited from Carpenter (1912) (citation no. 24 on p. 78), to whom the original data should be attributed. Beier (1948) also incorrectly stated England as the country of origin; the correct locality is Ireland.

^54^ Pseudoscorpion as *Chelifer nodosus* Schrank, 1803.

^55^ Pseudoscorpion as *Chernes reussii* (C.L. Koch, 1843), a junior synonym of *L. nodosus*, synonymised by Simon (1879).

^56^ Pseudoscorpion as Chelifer (Chernes) nodosus.

^57^ Pseudoscorpion as *C. cancroides* in Stephens (1910), but in Kew (1916) redefined as Chelifer (C.) godfreyi, later synonymised with *L. savignyi* by Muchmore (1975).

^58^ Pseudoscorpion as Chelifer (C.) godfreyi, later synonymised with *L. savignyi* by Muchmore (1975). Beier (1948) incorrectly stated England as the country of origin, whereas the correct locality is Ireland.

^59^ Host as *Musca vicina* Macquart, 1851. Pseudoscorpion as *Muscichernes katoi* Morikawa, 1960, a junior synonym of *L. savignyi*, synonymised by Harvey (1987).

^60^ Pseudoscorpion as *Pycnochernes* Beier, 1932, a junior synonym of *Lamprochernes*, synonymised by Muchmore (1975).

^61^ Pseudoscorpion as *Obisium muscorum*.

^62^ Pseudoscorpion as *C. cancroides*; in Beier (1948) as *Neochernes sanborni* (Hagen, 1869). Muchmore (1971) stated that this record cannot be identified as *Chernes sanborni* Hagen, 1868, and is therefore treated here as Not specified.

^63^ In the original paper, the phoront is mentioned only as a pseudoscorpion; in Beier (1948), it is given as *N. sanborni*. Muchmore (1971) stated that this record cannot be identified as *Chernes sanborni* Hagen, 1868, and is therefore treated here as Not specified.

^64^ Pseudoscorpion as *Dinocheirus sicarius* Chamberlin, 1952, a junior synonym of *Dinocheirus serratus* (Moles, 1914), synonymised by Muchmore (1997).

^65^Beier (1948) mentioned the host only as Diptera, whereas in the original data, it is specified as a muscid (Ellingsen 1913). Pseudoscorpion as *Chelifer nodosus* in the original paper from Gold Coast, West Africa (present-day Ghana).

^66^ Pseudoscorpion as *Chernes nodosus*. Data on phoresy based on Plate XXX.

^67^ Pseudoscorpion as Chelifer (C.) godfreyi, later synonymised with *L. savignyi* by Muchmore (1975).

^68^ Pseudoscorpion as *Chernes scorpioides* (Hermann, 1804).

^69^ The paper also includes data from experiments; however, only field data were considered here.

^70^ Pseudoscorpion *Semeiochernes armiger* Balzan, 1892 – the author and year should be given in parentheses.

^71^ Pseudoscorpion as *Semeiochernes militaris* Beier, 1932; in Aguiar and Bührnheim (1998b: 458), a host is added and the species is given as *S. armiger*.

^72^ Data mentioned in the Addendum.

^73^ Host as *Omphrale fenestralis* (Linnaeus, 1758).

^74^ Host as *Leptocera sylvatica* [sic] (Meigen, 1830) (should be *Leptocera sylvatica*).

^75^ Host as *Hoplites* [sic] bispinosus Macq. (should be *Hoplistes bispinosus* (Wiedemann, 1830)). Pseudoscorpion not identified in the original paper; treated as *L. nodosus* in Beier (1948).

^76^ The host family as Stratiomydae [sic] (should be Stratiomyidae).

^77^ Pseudoscorpion as Chelifer (Chernes) nodosus in Berland (1932).

^78^ Host given as “Serphid fly”, a misspelling referring to a syrphid fly (hoverfly).

^79^ Host as *Musca larvarum* Linnaeus, 1758 (Muscidae), now *Exorista larvarum* (Tachinidae). Pseudoscorpion as *C. cancroides* in the original paper; treated as a misidentification in WPC (2026).

^80^ Host as “Dexidae” (and “Dexiidae”) refers to the tachinid subfamily Dexiinae (Tachinidae), not to Dixidae; these spellings represent historical variants of Dexiinae rather than a misidentification. Pseudoscorpion as *Chelanops pallipes* Banks, 1893.

^81^ Pseudoscorpion given as *C. cimicoides* = *C. hahnii* in Wagner (1892); in Berland (1932) as Chelifer (Chernes) nodosus. In WPC (2026), this record is treated as a misidentification and referred to *L. nodosus*.

^82^ Pseudoscorpion as *Chelifer geoffroyi* Leach, 1817, a junior synonym of *Chernes cimicoides*, synonymised by Simon (1879). In WPC (2026), this record is treated as a misidentification and referred to *L. nodosus*.

^83^ Pseudoscorpion as genus *Obisium* Illiger, 1798; as *Syarinus obscurus* (Banks, 1893) in Muchmore (1971).

^84^ Pseudoscorpion as *Dactylochelifer latreillii* (Leach, 1817).

^85^ Host as *Chrysomyza demandata* (Fabricius, 1798).

^86^ Host as *Ulidia demandata* (Fabricius, 1798). Pseudoscorpion as *Chelifer corallinus* Loew, 1845, later synonymised with *L. nodosus* by Tömösváry (1883).

^87^ Host as *Chloria demandata* (Fabricius, 1798). Pseudoscorpion as *Chelifer hahnii* C.L. Koch, 1839, treated as a misidentification in WPC (2026) and referred to *L. nodosus*.

^88^ Host as *Physiphora demandata* (Fabricius, 1798).

^89^ Pseudoscorpion as “probably Chernetidae”, treated here as Not specified.

^90^ Host as *Chrysomyza aenea* (Fabricius, 1794).

^91^ Pseudoscorpion as *Chelifer wideri* C.L. Koch, 1843, treated as a misidentification in WPC (2026) and referred to as *L. nodosus*.

^92^ Pseudoscorpion as *Chelifer loewi* – nomen nudum.

^93^ Pseudoscorpion as chelifers in Hagen (1867, 1868); treated as *Neochernes sanborni* by Muchmore (1971), currently as *Chernes sanborni*.

^94^ Pseudoscorpion as *Chernes santorni* [sic] (should be *Chernes sanborni*).

^95^ Pseudoscorpion females found at the bottom of the Malaise trap were not considered phoretic.

^96^ Host as a fly; pseudoscorpion as *Chernes nodosus*. In Beier (1948: 442), another record of *L. nodosus* on *Calliphora* is mentioned, but this could not be found in the original paper.

^97^ Several fly species are listed under Diptera.

^98^ Pseudoscorpion as *Chelifer nodosus*. In Beier (1948) and WPC (2026), the record referred to *Lamprochernes godfreyi*. Chelifer (Chernes) godfreyi is a junior synonym of *Lamprochernes savignyi*, synonymised by Muchmore (1975).

^99^ Pseudoscorpion as Chelifer (Chernes) godfreyi Kew, 1911 on flies. Chelifer (Chernes) godfreyi is a junior synonym of *Lamprochernes savignyi*, synonymised by Muchmore (1975).

Data about *Chernes nodosus* from Kew (1911) are just citations of other papers. Beier (1948) treats these under *L. nodosus*, but these records are based solely on previously published sources and do not constitute new data.

^100^ The records from Beier (1946, 1948) for Sudan were merged into a single record (one female from a fly trap, February).

^101^ Pseudoscorpion as *Chelifer scorpioides* Hermann, 1804.

### Fossil records

The fossil data were compared with Poinar et al. (1998) and supplemented with three studies published subsequently. Table 2 includes, where provided in the original publications, information on host and phoront taxa, amber type, and reference. Missing data from published records are marked as “Not specified” in Table 2. Notes on discrepancies and differences among records are presented in Table 2. Altogether, five pseudoscorpion records were documented, of which three were identified to species or genus level. These phoronts were attached to the legs of five dipteran taxa representing three families, with some hosts remaining unidentified; only two dipteran species could be identified to species level (Table 2). Regarding amber provenance, most records originate from Baltic amber (Table 2), indicating that phoretic associations between pseudoscorpions and their dipteran hosts were already established by at least the Paleogene.

**Table 2.** Fossil records of phoresy between Pseudoscorpiones and Diptera.

Host taxa	Phoront taxa		Amber type	References
** Limoniidae **				
*Trentepohlia immemorata* Podenas & Poinar, 2001	Cheliferidae	*Hysterochelifer manpauch* Córdova-Tabares, Riquelme & Villegas-Guzmán, 2024	Mexican amber	Córdova-Tabares et al. (2024)
Not specified^1^	Not specified	Not specified	Baltic amber	Schlee and Glöckner (1978)
** Tipulidae **				
Not specified	Garypinidae	*Garypinus electri* Beier, 1937	Baltic amber	Judson (2004)
** Xylophagidae ** ^2^				
*Chrysothemis speciosa* Loew, 1850	Not specified	Not specified	Baltic amber	Ross (1997)
Not specified^3^	Cheliferidae	*Electrochelifer* Beier, 1937	Bitterfeld amber	Ahrens et al. (2019)

Notes: ^1^ In Poinar et al. (1998) as Tipulidae and Chernetidae; ^2^ Host family originally as Rachiceridae; ^3^ Host only as probably Nematocera.

## Conclusion

In summary, following a revision of published data and the addition of new European records, pseudoscorpions were recorded phoretically on dipteran hosts belonging to 74 species (59 genera) from 30 families, with the majority of records associated with *Musca domestica* (Muscidae). Although 47 records are identified only at the level of Diptera, pseudoscorpions participating in phoretic associations with Diptera represent 39 species (21 genera) from seven families, most of which belong to the genus *Lamprochernes*. However, these data are likely biased, as earlier studies predominantly reported *L. nodosus* attached to the house fly, a pattern further reinforced by the extensive redeterminations of specimens by Beier (1948). This taxonomic uncertainty complicates the interpretation of historical data and underscores the need for careful reassessment of species identities in phoretic records. Geographically, the records originate from 44 countries, although some data were reported only at the continental level. Fossil evidence from amber further indicates that pseudoscorpion–Diptera phoretic associations represent a long-standing ecological interaction, already established by at least the Paleogene and persisting into the present day. Compared with Poinar et al. (1998), the number of dipteran hosts increased by 37 species (27 genera) and 13 families, while the number of pseudoscorpion phoronts increased by 16 species (five genera) and three families. This synthesis provides the most comprehensive overview to date of pseudoscorpion–Diptera phoretic associations and establishes a robust framework for future taxonomic, ecological, and evolutionary studies.

## Additional information

### Use of AI

No use of AI was reported.

### Author ORCIDs

Jana Christophoryová https://orcid.org/0000-0002-3746-1367

Vincent Mathy https://orcid.org/0000-0002-8448-3112

Christoph Hörweg https://orcid.org/0000-0002-8208-0758

Lucia Vičanová https://orcid.org/0009-0005-9558-8437

### Data availability

All of the data that support the findings of this study are available in the main text.
